# Use of “Non-Traditional” Animal-Assisted Interventions in Children: A Scoping Review

**DOI:** 10.3390/ejihpe16100160

**Published:** 2026-10-09

**Authors:** Margaret E. Gruen, Navika Mittal, Peggy Gross, Mariam A. Hamad, Anika R. Kadumpalli, Rebecca J. Harney, David C. Dorman

**Affiliations:** 1 College of Veterinary Medicine, North Carolina State University, Raleigh, NC 27607, USA; nmittal3@ncsu.edu (N.M.); mahamad@ncsu.edu (M.A.H.); david_dorman@ncsu.edu (D.C.D.); 2 William Rand Kenan, Jr. Library of Veterinary Medicine, University Libraries, North Carolina State University, Raleigh, NC 27607, USA

**Keywords:** animal-assisted interventions, animal-assisted therapy, children, therapy animals

## Abstract

Animal-assisted interventions (AAI) are increasingly used to support pediatric health, education, and psychosocial well-being. While most research has focused on dogs and horses, a growing body of literature has examined the use of other less traditional animal species. This scoping review mapped the existing evidence on non-traditional AAI in children and adolescents. Using a Population–Concept–Context framework, studies involving participants aged ≤18 years and non-traditional AAI were identified and reviewed. Data were extracted on intervention characteristics, animal species, populations, settings, outcomes, and study design. Forty-eight studies met inclusion criteria. Interventions involved diverse species, including dolphins, rabbits, guinea pigs, donkeys, goats, cows, cats, fish, reptiles, insects, turtles, giant snails, and elephants. Studies were conducted primarily in schools, therapy centers, farms, hospitals, and residential settings. Common populations included children with autism spectrum disorder, Down syndrome, developmental disabilities, anxiety, trauma-related experiences, and other health conditions. Outcomes are most frequently related to social communication, emotional well-being, anxiety, stress, self-esteem, cognition, and physical functioning. Although favorable outcomes were frequently reported, the studies varied considerably in design, populations, interventions, and outcome measures. Because the methodological quality and risk of bias of included studies were not formally assessed, conclusions regarding the efficacy or effectiveness of non-traditional AAI cannot be drawn from this review. The findings instead characterize the scope and diversity of the existing literature and identify areas for further investigation. The evidence base is characterized by small samples, methodological heterogeneity, and relatively few controlled studies, highlighting the need for more rigorous research.

## 1. Introduction

Animal-assisted interventions (AAI) and animal-assisted services (AAS) encompass a range of approaches that intentionally incorporate animals into human services to support health, well-being, and functioning (Dixon et al., 2025). Within pediatric contexts, AAI typically includes animal-assisted therapy (AAT), defined as goal-directed, structured interventions delivered by qualified professionals, and animal-assisted activities (AAA), which are generally less formal and focus on engagement, enrichment, or well-being (IAHAIO, 2018; Rogers, 2024). Activities may include petting, grooming, walking, playing with, reading to, or interacting with an animal. Reported benefits span emotional (e.g., reduced anxiety, improved mood, enhanced self-esteem), social (e.g., improved communication, cooperation, and social engagement), cognitive (e.g., attention, memory, and learning), and physical (e.g., balance, coordination, and motor skills) domains (Mota-Rojas et al., 2026). Interest in pediatric AAI has increased substantially over the past two decades due to their perceived acceptability, feasibility, and potential benefits for children with diverse medical, developmental, and psychosocial needs (Doan et al., 2023). Terminology remains a barrier to understanding and studying AAI (Dixon et al., 2025). Contemporary AAI frameworks emphasize not only human outcomes but also the welfare of participating animals, recognizing that responsible implementation of interventions requires attention to the well-being of both human and animal participants (IAHAIO, 2018).

Research evaluating human–animal interactions (HAI) has generated several theoretical frameworks intended to explain the development and benefits of human–animal relationships. These frameworks include the biophilia hypothesis, which suggests that humans have an innate affinity for nature; the social support hypothesis, which postulates that animals act as buffers against stress; and the anthropomorphism hypothesis, which attributes human traits to non-humans (Beck, 2014; Peeters et al., 2026). Proposed mechanisms include neuroendocrine stress reduction, nonjudgmental social support, enhanced motivation and engagement, facilitation of social interaction, emotional co-regulation, and reciprocal human–animal relational processes (Beetz et al., 2012; Leconstant & Spitz, 2022; Peeters et al., 2026; Virués-Ortega & Buela-Casal, 2006). However, whether these mechanisms generalize across animal species remains unclear.

The pediatric AAI literature encompasses a broad range of populations, including hospitalized children, children with autism spectrum disorder (ASD), neurodevelopmental disabilities, trauma exposure, and chronic health conditions (Brandão et al., 2025; Feng et al., 2021; Harkness & Moorhead, 2025; Hoagwood et al., 2017; Lavín-Pérez et al., 2023; Moe et al., 2025; Rehn et al., 2023; Trzmiel et al., 2019; Waite et al., 2018; Xiao et al., 2024; Zhang et al., 2021). Studies have reported outcomes across multiple domains, including anxiety and pain reduction, social communication, emotional regulation, attention, and motor functioning. Dogs represent the most frequently studied and utilized species in pediatric AAI, followed by horses used in equine-assisted services. Pediatric AAI programs are implemented in hospitals, outpatient clinics, schools, and community settings (Koukourikos et al., 2019). However, the literature is highly heterogeneous with respect to intervention characteristics, animal species, frequency, settings, outcome measures, and study design. In addition, inconsistent use of terminology—particularly the interchangeable use of AAT and AAA—has been identified as a challenge for evidence synthesis and interpretation (Hill et al., 2019).

Several systematic reviews and meta-analyses have examined the effectiveness of pediatric AAI and generally report positive, though variable, effects across emotional, social, physical, and pain-related outcomes (Brandão et al., 2025; Charry-Sánchez et al., 2018; Feng et al., 2021; Hardy & Weston, 2020; Hoagwood et al., 2017; Lavín-Pérez et al., 2023; Moe et al., 2025; Rehn et al., 2023; Stergiou et al., 2017; Trzmiel et al., 2019; Waite et al., 2018; Xiao et al., 2024; Zhang et al., 2021). The strongest evidence has been observed for short-term reductions in pain and anxiety, particularly in hospital and procedural settings (Calcaterra et al., 2015; Waite et al., 2018). Reviews focusing on developmental, behavioral, and mental health outcomes, including studies involving children with ASD, suggest potential benefits for social communication, adaptive behavior, and emotional functioning; however, conclusions are limited by small sample sizes, variability in intervention protocols, inconsistent outcome measures, and risk of bias (Trzmiel et al., 2019). Across reviews, authors consistently call for greater methodological rigor, clearer reporting of intervention components, standardized terminology, and improved study design.

Despite the growing evidence base, most reviews have focused primarily on interventions involving dogs and horses. The predominance of dogs and horses likely reflects children’s familiarity with these species, established training and certification systems, broad public acceptance, extensive historical use in therapeutic settings, and the availability of an established evidence base. Species differ substantially in their behavioral repertoires, social tendencies, communication styles, sensory characteristics, and opportunities for human interaction. Consequently, potential mechanisms underlying responses observed during AAI may differ across species, potentially influencing intervention goals, participant engagement, and outcomes (Dixon et al., 2025; Peeters et al., 2026). The use of different animal species may provide distinct sensory, behavioral, and relational experiences that influence therapeutic processes and outcomes in children. There may also be advantages to using different animal species including availability of animals in schools or farms, cultural preferences, lower costs, human allergy concerns, and facility limitations that limit the use of dogs or horses. Understanding how species characteristics contribute to intervention effects is an important but understudied area of pediatric AAI research despite the increasing use of non-traditional species in pediatric settings.

Given the breadth, diversity, and methodological variability of the literature, a scoping review methodology is appropriate for mapping the field rather than evaluating intervention effectiveness. Although no standardized definition of non-traditional AAIs exists, this review operationally defines non-traditional species as those other than dogs and horses because these species account for most of the pediatric AAI literature. Our focus on children recognizes that developmental factors, including imaginative engagement, play-based learning, emotional responsiveness, and the formation of social relationships, may make pediatric populations uniquely responsive to animal-assisted interventions (Purewal et al., 2017). By systematically mapping the extent and nature of pediatric non-traditional AAI research, this review aims to inform future research priorities, support clearer conceptualization of interventions, and guide the development of more rigorous studies in this emerging area. As interest in animal-assisted interventions expands beyond traditional canine and equine models, a comprehensive understanding of the populations, settings, species, and outcomes associated with non-traditional interventions is needed to inform clinical practice, future research, and animal welfare considerations.

## 2. Materials and Methods

### 2.1. Objectives

This scoping review was conducted in accordance with the Preferred Reporting Items for Systematic Reviews and Meta-Analyses Extension for Scoping Reviews (PRISMA-ScR) guidelines (Tricco et al., 2018). A review protocol (“Use of ‘Non-traditional’ Animal-Assisted Therapy in Children”) was developed a priori, deposited in the Open Science Framework (OSF) before the review commenced, and subsequently registered in OSF. Several modifications were made to the original protocol while conducting the review. First, the eligibility criteria were revised prior to exclude interventions involving domesticated horses, in addition to those involving dogs. This change was made because additional scoping of the literature indicated that both dogs and horses are well-established species within the AAI literature. This change was consistent with our original objective of the review, namely to characterize the less extensively studied use of other animal species. We also expanded the Concept domain beyond studies evaluating participant outcomes to include studies examining potential intervention mechanisms, participant experiences, implementation processes, and physiological responses associated with AAI. This modification was made to better align the eligibility criteria with the mapping purpose of a scoping review and to capture evidence relevant to understanding how non-traditional AAI are delivered and studied. Both substantive eligibility modifications were made before execution of the formal database searches and before study screening began; thus, they were applied prospectively and consistently to all records identified by the review search. Finally, one additional member of the research team (RJH) was added during the review process. These protocol modifications and their rationale are reported here to provide transparency regarding differences between the original protocol and the completed review. Table S1 provides the completed PRISMA checklist for this study.

The objectives of this scoping review were to (1) identify and characterize the range of non-traditional AAIs used with children and adolescents; (2) characterize the non-traditional animal species represented in these interventions; (3) describe the settings in which these interventions have been implemented; (4) characterize the pediatric populations and conditions studied; (5) describe the outcome domains represented in the literature; and (6) identify methodological characteristics and evidence gaps in the existing literature. Consistent with the exploratory aims of a scoping review, eligible studies were not limited to evaluations of intervention effectiveness and could include investigations of intervention mechanisms, implementation approaches, participant experiences, and physiological responses associated with AAIs.

### 2.2. Eligibility Criteria

Study eligibility was guided by the Population–Concept–Context (PCC) framework.

- Population: Children and adolescents aged 18 years or younger, with or without diagnosed medical, developmental, behavioral, or psychosocial conditions.
- Concept: Animal-assisted interventions, including animal-assisted therapy (AAT), animal-assisted activities (AAA), animal-assisted education (AAE), and related studies examining intervention processes, mechanisms, participant experiences, physiological responses, or implementation characteristics involving animal species other than domesticated dogs or horses.
- Context: Any healthcare, educational, community, residential, or therapeutic setting.

Empirical studies of any design were eligible for inclusion, including randomized controlled trials, non-randomized studies, observational studies, qualitative studies, mixed-methods studies, implementation studies, and mechanistic or physiological studies examining processes or responses associated with animal-assisted interventions. Reviews, editorials, commentaries, conference abstracts, protocols, and animal-only studies were excluded. Only peer-reviewed studies published in English were considered.

### 2.3. Information Sources and Search Strategy

The search strategy was developed by an information specialist (PG) with expertise in systematic review methodology and literature searching. Searches were conducted without restrictions on publication year to comprehensively capture the available literature. Searches were conducted in MEDLINE (PubMed), PsycINFO, CINAHL, Scopus, and Web of Science. Searches were developed over the course of several weeks and conducted on 13 February 2026. This date refers to execution of the final searches across the specified databases. Study screening, full-text eligibility assessment, data extraction, and verification were subsequently conducted as separate stages of the review process, as described below. Authors were not contacted for missing information.

The search strategy combined controlled vocabulary and keyword terms related to animal-assisted interventions (e.g., “animal-assisted therapy,” “animal-assisted activities,” “animal-facilitated,” and “therapy animal”) and pediatric populations (e.g., “child,” “adolescent,” and “pediatric”). Search syntax was adapted as necessary for each database. The complete electronic search strategies, including the database-specific syntax used for each database, are provided in Table S2.

### 2.4. Study Selection, Data Extraction, and Synthesis

All identified records were imported into Covidence and duplicate records were removed. Titles and abstracts were independently screened by two reviewers against the eligibility criteria. Full-text articles were retrieved for potentially eligible records and independently assessed by two reviewers. Table S3 provides the full list of studies that were excluded at the full text review step and the reason for their exclusion. Disagreements were resolved through discussion and, when necessary, consultation with a third reviewer.

For each included study, data were extracted using a standardized form. Extracted information included bibliographic characteristics, study design, participant characteristics, intervention characteristics, animal species, setting, outcome measures, and key findings (Table 1). Data extraction was completed by one reviewer and verified by a second reviewer. ChatGPT (OpenAI, GPT6-Pro) also assisted with data extraction, structuring extracted information, and generating preliminary study summaries. ChatGPT suggested preliminary organization of the extracted information, including exploration of possible groupings; however, the evidence-stream definitions and final assignment of studies were determined by the authors and verified against the extracted data and original sources. All extracted data, interpretations, and final analyses were independently verified against the original source documents by the review team.

Consistent with the objectives of a scoping review, which were to map the extent, characteristics, and range of the available literature rather than determine intervention effectiveness, formal critical appraisal or risk-of-bias assessment of individual studies was not conducted (Veroniki et al., 2025). Accordingly, study findings were summarized descriptively and data synthesis focused on mapping the extent, nature, and key characteristics of the evidence base, identifying patterns across intervention types, populations, settings, animal species, and outcome domains, and highlighting gaps to inform future research. Because the review encompassed studies addressing different aspects of AAIs, evidence was characterized according to its primary focus. Studies were distinguished as examining participant outcomes, potential mechanisms of action, implementation or feasibility, participant experiences, or animal welfare. These categories were used to describe the nature of the evidence and were not intended as mutually exclusive classifications, as some studies addressed more than one domain.

## 3. Results

**Figure 1:**
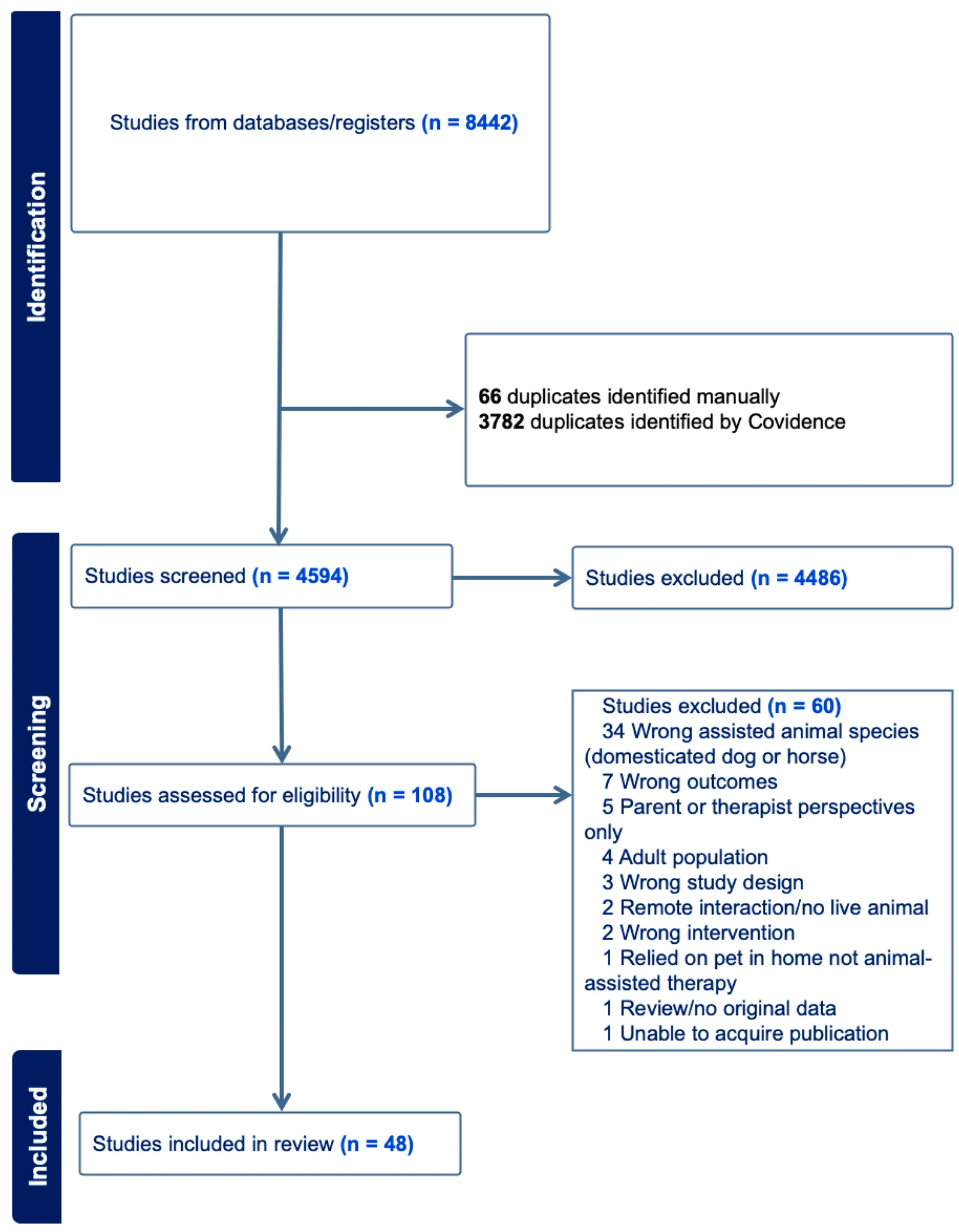
PRISMA diagram describing search workflow.

A total of 48 studies met the inclusion criteria (Figure 1). Studies were published between 1995 and 2026 and were conducted across Europe, North America, Asia, Australia, and the Middle East, demonstrating the international interest in non-traditional animal-assisted interventions.

### 3.1. Overview of Results

The evidence base included randomized controlled trials, quasi-experimental studies, observational studies, qualitative studies, mixed-methods studies, case studies, and mechanistic investigations. The most frequently studied populations included children with autism spectrum disorder (ASD), developmental disabilities, Down syndrome, anxiety-related conditions, chronic health conditions, and psychosocial challenges. Several studies also involved typically developing children in educational settings. Most studies evaluated intervention outcomes, whereas a smaller number focused on physiological mechanisms, implementation processes, stakeholder perceptions, or human–animal relationship development.

The included studies examined a broad range of non-traditional animal species (Table S1). Dolphin-assisted interventions accounted for the largest proportion of included studies (*n* = 15), followed by rabbit-assisted (*n* = 7) and guinea pig-assisted (*n* = 4) interventions. Beyond dolphins, rabbits, and guinea pigs, the evidence base included donkey-assisted (*n* = 3), fish/aquatic (*n* = 3), cat-assisted (*n* = 2), invertebrate-based (*n* = 2), and several mixed farm-animal (*n* = 5) and multi-species programs (*n* = 4), illustrating substantial species diversity. Several studies also evaluated interventions involving multiple species implemented in farm, educational, therapeutic, and community-based settings (Table 2).

The included studies addressed several distinct but overlapping evidence streams (Table 3). Most examined outcomes in participating children, while smaller bodies of literature addressed potential mechanisms of action, implementation and feasibility, participant or caregiver experiences and human–animal relationships, and animal welfare. Some studies contributed to more than one evidence stream. These categories distinguish the types of questions addressed by the literature and should not be interpreted as equivalent evidence regarding intervention effectiveness. Table S5 provides additional details regarding the characteristics of non-traditional animal species used in children.

### 3.2. Dolphin-Assisted Interventions

Dolphin-assisted interventions represented the largest body of evidence (*n* = 15 studies) and included intervention studies (Breitenbach et al., 2009; Dilts et al., 2011; R. E. Griffioen & Enders-Slegers, 2014; R. Griffioen et al., 2019; Hernández-Espeso et al., 2021; Kreiviniene & Kleiva, 2017; MdYusof & Chia, 2012; Salgueiro et al., 2012; Stumpf & Breitenbach, 2014), mechanistic investigations (Matamoros et al., 2020; Matamoros et al., 2022; Moreno Escobar et al., 2023; Moreno Escobar et al., 2024), an experimental reinforcement study (Nathanson, 2007), and implementation-focused research (Kreiviniene & Aciene, 2026).

Intervention studies primarily involved children with ASD, Down syndrome, cerebral palsy, developmental disabilities, and communication impairments. Reported outcomes included improvements in communication, social interaction, verbalization, adaptive behavior, self-confidence, and selected social-emotional domains. However, findings were mixed, with several studies reporting limited effects, improvements restricted to specific outcome measures, or benefits that were not consistently supported across informants or comparison groups (Breitenbach et al., 2009; Hernández-Espeso et al., 2021; Salgueiro et al., 2012; Stumpf & Breitenbach, 2014).

A notable feature of the dolphin literature was the presence of several mechanistic and technology-focused studies. These investigations used electroencephalography (EEG), bioacoustic analyses, machine-learning approaches, and physiological monitoring to explore potential neurophysiological responses associated with dolphin-assisted therapy (Matamoros et al., 2020; Matamoros et al., 2022; Moreno Escobar et al., 2023; Moreno Escobar et al., 2024). Although generally exploratory and based on small samples, these studies suggested that dolphin-assisted interventions may be associated with measurable changes in brain activity, attention, physiological arousal, and behavioral engagement. Nathanson (2007) examined reinforcement mechanisms and reported that an animatronic dolphin functioned as an effective reinforcer, highlighting the possibility that some observed benefits may be attributable to motivational or reinforcement-related processes rather than direct interaction with live dolphins.

Additional implementation and perception studies explored family experiences, parental perspectives, and the integration of dolphin-assisted therapy within broader healthcare systems (Kreiviniene & Kleiva, 2017; Kreiviniene & Aciene, 2026). Collectively, the dolphin literature was characterized by substantial methodological diversity, encompassing effectiveness, mechanistic, and implementation-focused research. No other non-traditional species generated a comparable volume or diversity of research.

### 3.3. Rabbit- and Guinea Pig-Assisted Interventions

Rabbit- and guinea pig-assisted interventions were implemented primarily in educational and classroom settings (Iváncsik et al., 2025; S. H. Kim, 2024; Molnar et al., 2020; O’Haire et al., 2013b; O’Haire et al., 2014; Sokal & Kahl, 2019) and among children with ASD (O’Haire et al., 2013a; O’Haire et al., 2014; O’Haire et al., 2015; Zagrai et al., 2024). Reported outcomes included reductions in anxiety, improvements in social interaction and communication, enhanced social skills, increased self-esteem, improved classroom functioning, greater school engagement, and improved well-being. Rabbit-assisted intervention studies reported reduced anxiety during the transition to school and among elementary school children, with particularly pronounced effects among students with higher baseline anxiety and those with special educational needs (Iváncsik et al., 2025; Molnar et al., 2020). Participation in rabbit-focused youth programs was also associated with positive youth development and improved well-being among school-aged children (Sokal & Kahl, 2019).

Several classroom-based guinea pig programs reported increased peer engagement, social approach behaviors, positive affect, and reductions in social withdrawal among children with ASD. Children with ASD displayed more social interactions and received more social approaches from peers in the presence of guinea pigs than in comparable toy-based conditions (O’Haire et al., 2013b). Classroom-based animal-assisted activities were also associated with improvements in teacher- and parent-reported social functioning (O’Haire et al., 2013b; O’Haire et al., 2014).

One mechanistic study found that guinea pigs appeared to function as social buffers, reducing physiological arousal during peer interactions among children with ASD (O’Haire et al., 2015). Rabbit-focused studies additionally examined animal welfare and human–animal relationships. Zagrai et al. (2024) reported improvements in communication, attention, compliance, and social behaviors among children with ASD while simultaneously monitoring rabbit welfare, finding evidence consistent with transient stress responses rather than sustained distress in the therapy animals. Molnar et al. (2020) also monitored rabbit behavior and reported no evidence of substantial stress during classroom participation. The rabbit literature also included one observational human–animal relationship study that showed that socially competent children demonstrated a particular interest in interacting with rabbits and that children’s social styles were reflected in their interactions with the animals (Wedl & Kotrschal, 2009).

### 3.4. Cat-Assisted Interventions

Two studies evaluated cat-assisted interventions (Boyer & Mundschenk, 2014; Demiralay & Keser, 2022). One study examined whether a live cat could facilitate social communication between children with language impairments and typically developing peers during speech-language therapy sessions (Boyer & Mundschenk, 2014). The second study evaluated the effects of a structured pet-therapy program using a therapy cat on stress and social anxiety among physically disabled children (Demiralay & Keser, 2022).

Both studies reported favorable findings. Boyer and Mundschenk (2014) found that two of three participants had more sustained verbal interaction and peer communication in the live-cat condition compared with toy-cat and preferred-activity conditions. Demiralay and Keser (2022) reported significant reductions in perceived stress and social anxiety among physically disabled children who participated in a seven-week cat-assisted intervention compared with controls. These studies reported favorable findings for social communication and psychological well-being, although the evidence base was limited to two studies.

### 3.5. Farm Animal and Livestock Interventions

Farm-based interventions ranged from structured therapeutic programs to educational and social-farming models. Farm animal studies included interventions involving donkeys, goats, cows, and mixed-species farm programs. Donkey-assisted intervention studies reported improvements in self-esteem, communication, emotional expression, motivation, relational skills, and selected educational outcomes (Corallo et al., 2023; De Rose et al., 2011; Dicé et al., 2017). Goat-assisted programs focused primarily on mood, quality of life, emotional well-being, and social engagement (Harada et al., 2019). Cow-assisted interventions represented a novel area of research. The cow-assisted pilot study reported favorable changes in self-esteem, emotional expression, social interaction, and openness toward dairy products while simultaneously evaluating animal welfare and economic feasibility (Torquati et al., 2025).

Mixed-species farm programs were implemented in educational, therapeutic, and social-farming contexts (Byström et al., 2022; Dolecheck et al., 2024; Geldhof et al., 2021; Novotná et al., 2025). These studies reported improvements in social communication, mentalization, emotion regulation, social functioning, self-esteem, and behavioral engagement among children with ASD, intellectual disabilities, and special educational needs. Educational programs involving direct contact with farm animals were additionally associated with improved knowledge retention and learning outcomes (Novotná et al., 2025).

### 3.6. Fish and Aquatic Species Interventions

Three studies examined goldfish-based interventions in healthcare settings (Sarman & Günay, 2023; Sarman & Tuncay, 2024; Yilmaz & Demir Acar, 2024). One study also evaluated aquatic turtle-assisted interventions alongside goldfish interventions during intravenous catheterization procedures (Sarman & Tuncay, 2024). All studies were conducted in hospital or outpatient clinical settings and focused on reducing distress associated with hospitalization or medical procedures.

The interventions primarily targeted anxiety, fear, pain, and psychological well-being among hospitalized children. Goldfish-assisted intervention studies reported significant reductions in state anxiety and fear, as well as improvements in emotional and psychological well-being among hospitalized children (Sarman & Günay, 2023). During intravenous catheterization, both goldfish and aquatic turtle intervention studies reported reduced procedural pain, anxiety, and fear compared with standard care (Sarman & Tuncay, 2024). Similarly, goldfish-assisted intervention studies in pediatric emergency departments reported reduced children’s fear levels and decreased fear related to nursing procedures and hospital equipment (Yilmaz & Demir Acar, 2024).

Collectively, these studies reported lower procedural distress, anxiety, fear, or pain among children exposed to fish- or aquatic-species interventions compared with control conditions. These findings identify distraction and comfort as possible intervention processes, but the present review did not formally appraise study quality or establish intervention effectiveness.

### 3.7. Exotic and Wildlife Species Interventions

Evidence involving elephants, reptiles, insects, and non-vertebrate species was limited but diverse. One elephant-assisted therapy study reported improvements in visual-motor integration among children with Down syndrome, although no significant effects on balance or postural control were observed (Satiansukpong et al., 2016). Reptile-assisted support programs using bearded dragons reported improvements in internalizing, externalizing, social, attention, and thought-related behaviors among female adolescents with emotional disturbances (Murry & Allen, 2012).

Insect-based educational studies reported increased self-esteem, reduced daily stress, and favorable changes in diurnal cortisol profiles among elementary school children (A. S. Kim et al., 2018). One study involving a Giant African land snail explored children’s attachment and caregiving relationships with a classroom animal and highlighted the educational and developmental value of interactions with non-traditional companion species (Hirschenhauser & Brodesser, 2023). These studies illustrate the range of therapeutic, educational, and psychosocial outcomes that have been examined using non-traditional species, although the evidence for individual species remains limited.

### 3.8. Multi-Species Programs

Several studies evaluated interventions involving multiple animal species (Conniff et al., 2005; Flynn et al., 2024; Locher et al., 2023; Mudaly et al., 2014; Sokal & Kahl, 2019). These programs were delivered in residential, educational, therapeutic, and community settings and frequently combined interactions with animals and broader psychosocial, educational, or recreational activities. Reported outcomes included improvements in emotional regulation, social functioning, communication, self-confidence, pain-related coping, and overall well-being. However, findings were not uniformly positive, as some studies reported favorable participant experiences without corresponding quantitative improvements.

### 3.9. Unknown-Species Programs

One intervention study evaluating the effectiveness of animal-assisted therapy in reducing anxiety symptoms among preschool children did not identify the animal species used (Tahan et al., 2022). The randomized controlled trial reported significant reductions in overall anxiety, separation anxiety, generalized anxiety, social phobia, and specific anxiety symptoms following animal-assisted therapy, although the species involved was not reported.

## 4. Discussion

### 4.1. Non-Traditional Species Expand the Conceptual Scope of Pediatric AAI

This review demonstrates that pediatric animal-assisted interventions extend well beyond the traditionally studied use of dogs and horses (Kovács et al., 2024). Across 48 studies, we found non-traditional species incorporated into educational, healthcare, rehabilitation, community, and farm-based programs involving children with diverse developmental, medical, behavioral, and psychosocial needs. Although dolphins represented the largest body of evidence, interventions involving rabbits, guinea pigs, donkeys, goats, fish, reptiles, insects, elephants, cattle, and mixed-species programs illustrate the considerable diversity of species currently being used in pediatric AAI.

The findings suggest that non-traditional species may broaden the conceptual and practical scope of pediatric AAI by providing distinct sensory, behavioral, and environmental experiences that may influence participant engagement and therapeutic processes. For example, small animals such as rabbits and guinea pigs provide opportunities for tactile interaction and may be readily integrated into classroom settings. Conversely, fish-based interventions generally involve passive observation and have been studied in relation to stress and anxiety outcomes. Dolphins, donkeys, and farm animals provide opportunities for experiences that are difficult to replicate with more traditional therapy animals. These differences may influence intervention goals, participant experiences, and implementation approaches.

The existing evidence base remains insufficient to determine whether specific species produce distinct therapeutic benefits or whether observed effects are primarily attributable to broader human–animal interactions. Nevertheless, the diversity of species represented in the reviewed literature suggests that non-traditional animals are not merely substitutes for dogs and horses. Rather, they appear to occupy unique roles within pediatric AAI that are shaped by intervention context, participant characteristics, and therapeutic objectives.

### 4.2. Species, Setting, and Intervention Goals Appear Closely Linked

The literature suggests that species selection is context-dependent and may reflect the practical, environmental, and therapeutic demands of specific intervention settings. For example, school-based interventions commonly involved rabbits and guinea pigs, which can be more easily housed in classrooms. In contrast, dolphins and elephants require highly specialized facilities, limiting their availability as therapy animals.

Our review found school-based interventions most often involved rabbits, guinea pigs, goats, insects, and mixed-species farm programs and were often classified as animal-assisted activities or animal-assisted education. These programs typically focused on social competence, emotional learning, classroom engagement, and educational outcomes. In contrast, dolphin-, donkey-, and elephant-assisted interventions, as well as some hospital-based animal-assisted therapies were generally delivered within specialized therapeutic or rehabilitation settings, and most targeted communication, developmental functioning, motor skills, or rehabilitation-related goals. Several interventions were implemented within green-care or social-farming environments, where interactions with multiple animal species were integrated into broader educational, recreational, and psychosocial programs. These settings highlight the extent to which animal-assisted interventions may be embedded within larger therapeutic or nature-based experiences rather than centered on a single animal species. In many other studies, animals were used within broader educational, recreational, rehabilitation, or psychosocial programs, making it difficult to isolate the unique contribution of the species from other intervention components.

Intervention duration also varied substantially across settings, ranging from single-session procedural interventions in hospitals to programs lasting several months or, in some cases, more than a year. Such variation further suggests that species selection, intervention intensity, and program objectives are closely interconnected.

Our findings suggest that some species are selected because they are easily integrated into existing environments. Thus, logistical and environmental constraints may shape the available evidence base as much as therapeutic considerations. Other factors, including species temperament, availability, and accessibility, may also influence species selection. Differences in animal husbandry requirements and accessibility influence costs, scalability, geographic availability, and equity of access to AAI programs that rely on certain nontraditional animal species. These factors may limit the transferability of findings across settings and influence the feasibility of implementing certain programs in routine practice. It remains unclear whether observed outcomes are attributable to species-specific characteristics or whether species are primarily selected because they fit the practical requirements of particular intervention settings.

Historical development of existing programs may also have contributed to availability bias within the literature. For example, the concentration of dolphin studies may partly reflect the historical popularity of dolphin-assisted therapy programs rather than evidence supporting superior effectiveness. Collectively, our findings suggest that non-traditional species are not interchangeable within pediatric AAI. Rather, species appear to be selected in ways that reflect the opportunities, constraints, and therapeutic objectives of specific educational, healthcare, rehabilitation, and community contexts.

Geographic patterns also appear to shape the available evidence. Although included studies were conducted across multiple world regions, research involving certain species was concentrated in certain countries or settings. For example, several of the mechanistic dolphin-assisted studies were conducted in Mexico, whereas rabbit-focused studies were represented in several European countries and guinea-pig studies were concentrated in Australia.

### 4.3. Social Communication and Emotional Well-Being as Dominant Outcome Domains

Despite substantial diversity in animal species, intervention settings, and participant populations, the most frequently reported outcomes involved social communication and emotional well-being. Reported outcomes generally clustered into five domains: social-communication, emotional-behavioral, cognitive-educational, physical, and physiological outcomes. Social communication was the most frequently reported outcome domain and often involved children with ASD, a population for whom social engagement and communication are often primary intervention goals. Studies involving dolphins, rabbits, guinea pigs, donkeys, and mixed farm-animal programs frequently reported improvements in communication, social engagement, peer interaction, verbalization, social approach behaviors, and relationship-building. Emotional and anxiety-related outcomes were reported across educational, healthcare, and therapeutic settings, indicating that these outcomes have been examined across a relatively broad range of intervention contexts. The recurrence of similar social and emotional outcome domains across markedly different animal species raises the hypothesis that common features of human–animal interaction—such as social engagement, emotional support, motivation, and opportunities for relationship-building—may contribute to observed responses. However, the present evidence does not establish these processes as causal mechanisms. Unlike social and emotional outcomes, favorable cognitive and educational outcomes were primarily reported in school-based interventions, suggesting that these outcomes may be more closely linked to intervention context and educational programming than to animal interaction alone. Physical outcomes were reported less frequently and were concentrated in interventions involving species capable of supporting movement-based, rehabilitation, or experiential activities, such as dolphins, elephants, and farm animals. This pattern further suggests that intervention goals may be shaped as much by the opportunities afforded by specific species and settings as by the animal itself. Physiological outcomes were comparatively uncommon and were primarily represented by mechanistic studies examining arousal, stress responses, or neurophysiological activity during animal-assisted interventions.

Although positive findings were common across outcome domains, relatively few studies reported null or adverse effects in detail. Given the predominance of small pilot studies and observational designs within the literature, caution is warranted when interpreting the consistency of reported benefits. The concentration of positive findings may partly reflect publication bias, selective outcome reporting, or limitations in study design.

Our findings suggest that social communication and emotional well-being represent the most frequently studied and most frequently favorably reported outcome domains in pediatric non-traditional AAI. The similarity of favorably reported outcomes across diverse species raises the possibility that therapeutic processes associated with human–animal interaction may be more influential than species identity alone. Whether these effects reflect species-specific characteristics or broader mechanisms of social engagement, emotional support, and motivation remains unclear and warrants further investigation.

### 4.4. Emerging Interest in Mechanisms, Animal Welfare, and Implementation

The presence of similar social and emotional outcomes across diverse animal species highlights the importance of understanding the mechanisms through which AAI may influence behavior. Unlike many reviews that focus primarily on intervention outcomes, this review also considered mechanistic investigations. A subset of studies focused primarily on understanding the mechanisms underlying AAI rather than evaluating clinical outcomes. These studies examined neurophysiological responses during dolphin-assisted therapy, emotion-recognition technologies, neurophysiological monitoring, behavioral coding systems, reinforcement processes, and participant experiences. Notably, most mechanistic investigations focused on dolphin-assisted interventions, leaving substantial uncertainty regarding whether similar processes operate across other non-traditional species.

Mechanistic studies highlighted potential pathways through which AAI may influence behavior and functioning, including increased social engagement, changes in physiological arousal, enhanced emotional processing, reinforcement effects, and alterations in neural activity. Qualitative investigations emphasized the importance of relationship-building, emotional safety, sensory experiences, and opportunities for choice and autonomy within animal-assisted programs, suggesting that participant experiences and therapeutic relationships may be important determinants of intervention success. Mechanistic investigations represented an emerging area of inquiry, particularly within dolphin-assisted interventions, but remain relatively uncommon across other species. As the evidence base develops, research examining potential mechanisms may help clarify the processes underlying responses observed during AAI, while appropriately designed outcome studies are needed to evaluate intervention effectiveness.

Several studies also examined implementation-related factors, including family experiences, participant perceptions, therapeutic relationships, and program delivery characteristics. These findings suggest that intervention success may depend not only on the animal species involved but also on the quality of implementation, participant engagement, and relational processes that occur during intervention. One of the clearest findings from the review process was that several potentially relevant studies did not evaluate pediatric animal-assisted interventions directly, yet addressed important contextual factors that may influence implementation and effectiveness. Parental, caregiver, educator, or stakeholder attitudes toward animal-assisted interventions rather than child outcomes have been the focus of other studies deemed outside the scope of the present review (Ferreira et al., 2016; Guérin et al., 2017; Gupta & Yadav, 2018; Nalini & Kumar, 2022). These studies explored parental acceptance of therapy animals in healthcare settings, preferences for specific animal species, perceptions of risks and benefits, and attitudes toward implementing AAI in educational environments. Collectively, they suggest that family acceptance, cultural attitudes, and stakeholder support may influence the feasibility and uptake of animal-assisted interventions but remain understudied within pediatric intervention research. Similarly, studies examining methods for selecting animals based on participant preference (Guérin et al., 2017; Gupta & Yadav, 2018) highlight the potential importance of matching intervention animals to individual child interests and needs, an issue that was rarely addressed in the included literature.

Several related areas of inquiry emerged during the review process that were not well represented within the included studies but may influence the implementation and effectiveness of pediatric animal-assisted interventions. For example, few studies evaluated animal welfare. Research examining stress tolerance and behavioral responses of therapy rabbits suggests that repeated interactions may affect animal well-being and should be considered when designing intervention protocols (Suba-Bokodi et al., 2022; Zagrai et al., 2024). Contemporary One Health and One Welfare frameworks emphasize that human and animal outcomes are interconnected and should be considered jointly when evaluating AAIs. Increasing attention has therefore been directed toward understanding not only therapeutic benefits for participants, but also the welfare implications for animals involved in intervention programs. Consideration of animal welfare may be particularly important when examining non-traditional species, as husbandry requirements, stress responses, and suitability for therapeutic settings may vary substantially among species.

Some studies involved interventions in which non-traditional species were combined with dogs, horses, or multiple animal species, making it difficult to determine the unique contribution of the non-traditional animal. This issue was also apparent among studies identified during screening but excluded from the review because dogs or horses constituted the primary therapy animals; some nevertheless incorporated exposure to other farm animals (e.g., Balluerka et al., 2014, 2015).

There is a need for investigations examining alternative delivery approaches, including virtual and psychoeducational animal-assisted programs, which may be particularly relevant for children who cannot safely interact with live animals because of medical, infection-control, or logistical constraints (Battaglini et al., 2026). Another largely unexplored area relates to the potential role of family-owned pets as sources of therapeutic benefit (Hart et al., 2018). This can be important since so-called pocket pets and nontraditional species are often present in the home environment. It remains unknown whether favorable outcomes reported in studies included in this review may also emerge through child interactions with household companion animals. Well conducted future studies using nontraditional species in homes can evaluate whether some therapeutic benefits attributed to structured interventions may also emerge through naturally occurring human–animal relationships.

One other interesting facet of the current review is that some studies examined the use of virtual animals. For example, one experimental study compared reinforcement effectiveness of real Atlantic bottlenose dolphins and an animatronic dolphin for children with disabilities (Nathanson, 2007). This study reported that the animatronic dolphin was as effective or more effective than dolphins as a reinforcer, especially for some profoundly disabled children. Additional studies evaluating the role of virtual animals in AAI remain needed since these approaches may expand access to AAIs for children who are unable to participate in traditional programs because of medical, geographic, financial, or infection-control barriers.

Future research should move beyond documenting outcomes to investigating the therapeutic processes through which AAIs exert their effects. A clearer understanding of these mechanisms may facilitate the development of more targeted interventions and help determine whether reported benefits are driven by species-specific characteristics, broader human–animal interaction processes, or features of the intervention context itself. Likewise, additional efforts to integrate animal welfare and implementation processes into AAI programs with nontraditional species is needed. Together, these gaps indicate that future research should move beyond questions of effectiveness alone to examine implementation factors, participant preferences, animal welfare, and alternative intervention models that may influence the feasibility, acceptability, and sustainability of pediatric animal-assisted interventions.

### 4.5. Strengths and Limitations of the Review

A major strength of this review is its broad examination of non-traditional AAIs across diverse pediatric populations, settings, and animal species. In addition to intervention studies, the review incorporated mechanistic and implementation-focused research, providing a more comprehensive understanding of the current evidence base.

However, several limitations should be acknowledged. Conclusions regarding the efficacy or effectiveness of non-traditional AAI cannot be drawn from this review. Consistent with the mapping purpose of the scoping-review design, we did not conduct a formal critical appraisal or study-level risk-of-bias assessment. Consequently, the methodological quality of individual studies and the extent to which their findings may have been influenced by bias were not evaluated. Because formal evaluation of intervention effects was not an objective of the prospectively designed scoping review, study-level risk-of-bias assessment was not incorporated into the review protocol or analytic framework. The absence of such appraisal means that the methodological quality of individual studies and the extent to which bias may have influenced their reported findings cannot be determined from this review. Several methodological features evident across the mapped literature warrant consideration when interpreting reported outcomes. These included small samples, uncontrolled or non-randomized designs, reliance on participant or caregiver-reported outcomes, heterogeneous outcome measures, and multicomponent interventions that often precluded attribution of observed changes specifically to an animal interaction. Publication bias was also not formally assessed, and studies reporting favorable outcomes may be more likely to be represented in the published literature. The predominance of favorable reported outcomes should therefore be interpreted cautiously and as a description of the existing literature rather than as evidence of the efficacy or effectiveness of non-traditional AAIs. The evidence map generated by this scoping review can inform the development of a prospectively specified systematic-review protocol focused on intervention efficacy/effectiveness, incorporating design-appropriate risk-of-bias assessment and, where appropriate, evaluation of certainty of evidence.

The review was restricted to English-language and peer-reviewed publications. Furthermore, the inclusion of multi-species interventions may have complicated study classification and synthesis. The literature on pediatric AAI is characterized by substantial heterogeneity in intervention terminology, outcome measures, and reporting practices, which may also have influenced synthesis and classification. Our review focused on pediatric populations which also represents a limitation. Future efforts to evaluate the use of nontraditional animal species with adult and senior populations or other sensitive subpopulations is also warranted. Finally, our review did not consider the role of pets since household pets do not meet the formal definition of an AAI.

## 5. Conclusions

This review demonstrates that pediatric AAIS extend well beyond dogs and horses and encompass a diverse range of non-traditional species used across educational, healthcare, rehabilitation, and community settings. Social communication and emotional well-being were the most frequently reported outcomes, although species selection appeared closely linked to intervention context and goals. Although favorable outcomes were reported across studies, substantial variation in study designs, populations, interventions, and outcome measures limits comparisons across the literature. In addition, because this scoping review did not include a formal critical appraisal or risk-of-bias assessment, the findings should not be interpreted as evidence of the efficacy or effectiveness of these interventions. Rather, the review maps the nature and extent of the existing evidence and identifies priorities for future research, including mechanisms of action, implementation processes, comparative research examining species and intervention characteristics, and more rigorous evaluation of intervention outcomes.

An important gap identified by this review was the limited evaluation of animal welfare across the pediatric AAI literature. Although several recent studies have begun incorporating behavioral and physiological assessments of participating animals, animal outcomes remain substantially underrepresented relative to human outcomes. Future pediatric AAI research should more consistently evaluate the experiences and welfare of participating animals alongside human outcomes, supporting a more comprehensive and ethically informed evidence base for pediatric AAIs.

## Figures and Tables

**Table 1:** Data extraction domains.

Category	Variables
Study Characteristics	Author, year, country
Participants	Age, diagnosis
Intervention	AAT/AAA/AAE
Animal Characteristics	Species
Setting	Hospital, school, community
Outcomes	Emotional, social, cognitive, physical

**Table 2:** Characteristics of Animal Species Used in Pediatric Non-Traditional Animal-Assisted Interventions (N = 48 Studies). Note: Species categories are not mutually exclusive because some studies involved multiple animal species. The review included 48 unique studies. Detailed study-level species classifications are provided in Table S4.

Species Category	Number of Studies	Pediatric Population	Setting	Intervention Type
Dolphins	15	ASD, Down syndrome, cerebral palsy, developmental and intellectual disabilities, communication disorders, and other special healthcare needs	Dolphin therapy centers, rehabilitation facilities	AAT, mechanistic/physiological studies, reinforcement studies, implementation/perception studies
Rabbits	7	ASD, preschool children, elementary school children, first-grade students, children with special educational needs	Schools, classrooms, educational settings	AAT, AAA, animal-assisted pedagogy, educational interventions, human–animal relationship studies, animal welfare studies
Guinea pigs	4	ASD, school-aged children	Schools, classrooms	AAA, classroom-based interventions, social skills interventions, mechanistic/physiological studies
Cats	2	Children with language impairments; physically disabled children	Schools, rehabilitation settings	AAT
Donkeys	3	Children with communication and rehabilitation needs, specific learning disorders, elementary school students	Rehabilitation centers, schools	AAT, AAE
Goats	1	Preschool and school-aged children	Schools, community settings	AAA, AAT
Fish and aquatic animals	3	Hospitalized children and children undergoing intravenous catheterization or emergency care procedures	Hospitals, outpatient clinics, emergency departments	AAA, animal-assisted interventions, distraction-based interventions
Elephants	1	Children with Down syndrome	School-based therapeutic program	AAT
Reptiles	1	Female adolescents with emotional disturbance	Support-group setting	Animal-assisted support
Invertebrates	2	Elementary school children	Classroom and educational settings	Educational interventions, classroom animal programs
Cattle	1	Adolescents removed from families	Social farming setting	AAA, AAE, AAT
Mixed farm-animal interventions	5	ASD, intellectual disability, special educational needs, developmental disabilities, learning difficulties, mental health concerns, and elementary school children	Farms, eco-centers, schools, therapeutic programs	AAT, AAE, nature- and animal-based interventions
Multi-species programs	4	Adolescents, children exposed to trauma, special education students, chronic pain populations	Residential facilities, schools, community and therapeutic settings	AAA, AAT, AAE, psychotherapy
Species not reported	1	Preschool children with anxiety	Clinical setting	AAT

**Table 3:** Evidence streams represented in studies of pediatric non-traditional AAIs.

Evidence Stream	Evidence Addressed	Species and Studies Contributing to Evidence Stream ^1^
Participant outcomes	Health, psychosocial, behavioral, educational, cognitive, physical, or physiological outcomes in participating children	Dolphins: Dilts et al. (2011); R. E. Griffioen and Enders-Slegers (2014); R. Griffioen et al. (2019); Hernández-Espeso et al. (2021); Kreiviniene and Kleiva (2017); MdYusof and Chia (2012); Salgueiro et al. (2012); Stumpf and Breitenbach (2014). Rabbits: Iváncsik et al. (2025); S. H. Kim (2024); Molnar et al. (2020); Sokal and Kahl (2019); Zagrai et al. (2024). Guinea pigs: O’Haire et al. (2013a, 2013b, 2014). Cats: Boyer and Mundschenk (2014); Demiralay and Keser (2022). Donkeys: Corallo et al. (2023); De Rose et al. (2011); Dicé et al. (2017). Goat: Harada et al. (2019). Mixed farm animals: Byström et al. (2022); Geldhof et al. (2021); Novotná et al. (2025). Cows: Torquati et al. (2025). Fish: Sarman and Günay (2023); Sarman and Tuncay (2024); Yilmaz and Demir Acar (2024). Turtles: Sarman and Tuncay (2024). Elephants: Satiansukpong et al. (2016). Reptiles: Murry and Allen (2012). Insects: A. S. Kim et al. (2018). Multiple species: Conniff et al. (2005); Locher et al. (2023); Mudaly et al. (2014). Unidentified species: Tahan et al. (2022).
Mechanisms	Physiological, neurological, behavioral, interactional, or reinforcement processes that may contribute to responses observed during AAI	Dolphins: Matamoros et al. (2020); Matamoros et al. (2022); Moreno Escobar et al. (2023); Moreno Escobar et al. (2024); Nathanson (2007); R. Griffioen et al. (2019). Guinea pigs: O’Haire et al. (2015).
Implementation and feasibility	Intervention delivery, feasibility, program structure, integration into services, recruitment, economic considerations, or sustainability	Dolphins: Kreiviniene and Aciene (2026). Donkeys: De Rose et al. (2011). Cows: Torquati et al. (2025). Multiple farm species: Locher et al. (2023).
Participant/caregiver experiences and human–animal relationships	Participant or caregiver perceptions and experiences, acceptability, relationships with animals, and meanings attributed to human–animal interactions	Dolphins: Breitenbach et al. (2009); Kreiviniene and Kleiva (2017); Kreiviniene and Aciene (2026). Rabbits: Wedl and Kotrschal (2009); Sokal and Kahl (2019). Giant African land snail: Hirschenhauser and Brodesser (2023). Mixed farm animals: Dolecheck et al. (2024). Multiple species: Flynn et al. (2024); Locher et al. (2023); Mudaly et al. (2014).
Animal welfare	Direct behavioral, physiological, or health-related assessment of animals participating in AAI	Rabbits: Molnar et al. (2020); Zagrai et al. (2024). Cows: Torquati et al. (2025).

^1^ Evidence streams are not mutually exclusive, and individual studies may contribute to more than one stream. Studies involving more than one eligible species may also appear under more than one species designation. Participant-outcome studies include studies reporting outcomes in participating children but should not be interpreted collectively as evidence of intervention efficacy or effectiveness. Mechanistic studies examine processes potentially underlying responses observed during AAI; implementation studies address intervention delivery and feasibility; experiential studies examine participant or caregiver perspectives and human–animal relationships; and animal-welfare studies directly evaluate outcomes in participating animals. Detailed characteristics of all included studies are provided in Table S2.

## Data Availability

All data are contained in this publication.

## Supplementary Materials

The following supporting information can be downloaded at https://www.mdpi.com/article/10.3390/ejihpe16100160/s1: Table S1: Preferred Reporting Items for Systematic reviews and Meta-Analyses extension for Scoping Reviews (PRISMA-ScR) Checklist; Table S2: Electronic Search Strategies Used; Table S3: List of Excluded Studies at Full Text Screening Step and Reason for Exclusion; Table S4: Animal Species Included in Pediatric Non-Traditional Animal-Assisted Intervention Studies and Characteristics of Their Use; and Table S5. Characteristics of Included Non-Traditional Animal-Assisted Intervention Studies Organized by Animal Species and Intervention Type.

## Abbreviations

The following abbreviations are used in this manuscript:

AAA	Animal-assisted activities
AAE	Animal-assisted education
AAI	Animal-assisted interventions
AAS	Animal-assisted services
AAT	Animal-assisted therapy
ASD	Autism spectrum disorder
DAT	Dolphin-assisted therapy
EEG	Electroencephalogram
HAI	Human-animal interactions
PCC	Population–Concept–Context
