# Supplementary material for: Use of “Non-Traditional” Animal-Assisted Interventions in Children: A Scoping Review
Source: Eur J Investig Health Psychol Educ. 2026 Oct 9;16(10):160. doi: 10.3390/ejihpe16100160 (PMC13703158; doi:10.3390/ejihpe16100160)
Supplement: Supplementary file 1 [file ejihpe-16-00160-s001.zip › ejihpe-4518286-supplementary.pdf]

Table S1: Preferred Reporting Items for Systematic reviews and Meta-Analyses extension for Scoping Reviews (PRISMA-ScR) Checklist

SECTION	ITEM	PRISMA-ScR CHECKLIST ITEM	REPORTED ON PAGE #
TITLE			
Title	1	Identify the report as a scoping review.	1 Title page
ABSTRACT			
Structured summary	2	Provide a structured summary that includes (as applicable): background, objectives, eligibility criteria, sources of evidence, charting methods, results, and conclusions that relate to the review questions and objectives.	Page 2 Abstract word limitation limited providing all details
INTRODUCTION			
Rationale	3	Describe the rationale for the review in the context of what is already known. Explain why the review questions/objectives lend themselves to a scoping review approach.	Pages 2 & 3 Introduction
Objectives	4	Provide an explicit statement of the questions and objectives being addressed with reference to their key elements (e.g., population or participants, concepts, and context) or other relevant key elements used to conceptualize the review questions and/or objectives.	Page 4 - Methods
METHODS			
Protocol and registration	5	Indicate whether a review protocol exists; state if and where it can be accessed (e.g., a Web address); and if available, provide registration information, including the registration number.	Page 3- Methods
Eligibility criteria	6	Specify characteristics of the sources of evidence used as eligibility criteria (e.g., years considered, language, and publication status), and provide a rationale.	Pages 4 & 5 - Methods
Information sources*	7	Describe all information sources in the search (e.g., databases with dates of coverage and contact with authors to identify additional sources), as well as the date the most recent search was executed.	Pages 4 & 5 - Methods
Search	8	Present the full electronic search strategy for at least 1 database, including any limits used, such that it could be repeated.	Table S2
Selection of sources of evidence†	9	State the process for selecting sources of evidence (i.e., screening and eligibility) included in the scoping review.	Page 5- Methods
Data charting process‡	10	Describe the methods of charting data from the included sources of evidence (e.g., calibrated forms or forms that have been tested by the team before their use, and whether data charting was done independently or in duplicate) and any processes for obtaining and confirming data from investigators.	Page 5 Methods
Data items	11	List and define all variables for which data were sought and any assumptions and simplifications made.	Page 5 Methods
Critical appraisal of individual	12	If done, provide a rationale for conducting a critical appraisal of included sources of evidence; describe the methods used and how this information was used in any data synthesis (if appropriate).	Not performed (scoping review) however

SECTION	ITEM	PRISMA-ScR CHECKLIST ITEM	REPORTED ON PAGE #
sources of evidence§			rationale given on Page 5 Methods
Synthesis of results	13	Describe the methods of handling and summarizing the data that were charted.	Page 5 Methods
RESULTS			
Selection of sources of evidence	14	Give numbers of sources of evidence screened, assessed for eligibility, and included in the review, with reasons for exclusions at each stage, ideally using a flow diagram.	Page 6 & Figure 1
Characteristics of sources of evidence	15	For each source of evidence, present characteristics for which data were charted and provide the citations.	Page 6; Table 2; Tables S4 & S5
Critical appraisal within sources of evidence	16	If done, present data on critical appraisal of included sources of evidence (see item 12).	See item 12
Results of individual sources of evidence	17	For each included source of evidence, present the relevant data that were charted that relate to the review questions and objectives.	Table 2; Tables S4 & S5
Synthesis of results	18	Summarize and/or present the charting results as they relate to the review questions and objectives.	Table 2
DISCUSSION			
Summary of evidence	19	Summarize the main results (including an overview of concepts, themes, and types of evidence available), link to the review questions and objectives, and consider the relevance to key groups.	Pages 8-11 also Pages 11-15
Limitations	20	Discuss the limitations of the scoping review process.	Page 15
Conclusions	21	Provide a general interpretation of the results with respect to the review questions and objectives, as well as potential implications and/or next steps.	Page 15
FUNDING			
Funding	22	Describe sources of funding for the included sources of evidence, as well as sources of funding for the scoping review. Describe the role of the funders of the scoping review.	Page 16

From: Tricco AC, Lillie E, Zarin W, O'Brien KK, Colquhoun H, Levac D, et al. PRISMA Extension for Scoping Reviews (PRISMAS

Table S2: Electronic Search Strategies Used.

Concept 1:
"Animal Assisted Therapy"[Mesh] OR "Therapy Animals"[Mesh] OR "Human-Animal Interaction"[Mesh] OR AAE[tiab] OR "animal assisted"[tiab] OR AAI[tiab] OR AAT[tiab] OR "Animal facilitated"[tiab] OR "therapy animal"[tiab] OR "therapy animals"[tiab] OR "pet program"[tiab] OR "pet programs"[tiab] OR "pet support"[tiab] OR "support animal"[tiab] OR "support animals"[tiab] OR "pet therapy"[tiab] OR "pet assisted"[tiab] OR "pet facilitated"[tiab] OR "animal therapy"[tiab] OR "animal program"[tiab] OR "animal programs"[tiab] OR "animal therapy"[tiab] OR "animal intervention"[tiab] OR "animal interventions"[tiab] OR "dolphin assisted"[tiab] OR "human pet"[tiab] OR (hamster[tiab] OR hamsters[tiab] OR ferrets[tiab] OR ferret[tiab] OR "guinea pigs"[tiab] OR "guinea pig"[tiab] OR gerbil[tiab] OR gerbils[tiab] OR rabbits[tiab] OR rabbit[tiab] OR hares[tiab] OR hare[tiab] OR cats[tiab] OR cat[tiab] OR cow[tiab] OR calf[tiab] OR goat[tiab] OR goats[tiab] OR fish[tiab] OR dolphin[tiab] OR dolphins[tiab]) AND (facilitated[tiab] OR therapy[tiab] OR interaction[tiab])
AND

Concept 2:
"Pediatrics"[Mesh] OR "Child"[Mesh] OR "Child, Preschool"[Mesh] OR "Adolescent"[Mesh] OR "Child"[tiab] OR children[tiab] OR preschool[tiab] OR preschoolers[tiab] OR adolescent[tiab] OR adolescents[tiab] OR pediatric[tiab] OR pediatrics[tiab] OR teen[tiab] OR teens[tiab] OR teenage[tiab] OR "teen agers"[tiab] OR teenagers[tiab] OR youth[tiab] OR youths[tiab])

NOT
"systematic review"[tiab] OR "systematic literature review"[tiab] OR "scoping review"[tiab] OR "systematic narrative review"[tiab] OR "systematic qualitative review"[tiab] OR "systematic evidence review"[tiab] OR "systematic quantitative review"[tiab] OR "systematic meta-review"[tiab] OR "systematic critical review"[tiab] OR "systematic mixed studies review"[tiab]

Concept 1 AND Concept 2 NOT Concept 3

Web of Science
Concept 1:
TS=("Animal Assisted" OR "Therapy Animals" OR "Human-Animal Interaction" OR AAE OR AAI OR AAT OR "Animal facilitated" OR "therapy animal" OR "therapy animals" OR "pet program" OR "pet programs" OR "pet support" OR "support animal" OR "support animals" OR "pet therapy" OR "pet assisted" OR "pet facilitated" OR "animal therapy" OR "animal program" OR "animal programs" OR "animal intervention" OR "animal interventions" OR "dolphin assisted" OR "human pet" OR ((hamster OR hamsters OR ferrets OR ferret OR "guinea pigs" OR "guinea pig" OR gerbil OR gerbils OR rabbits OR rabbit OR hares OR hare OR cats OR cat OR cow OR calf OR goat OR goats OR fish OR dolphin OR dolphins) AND (facilitated OR therapy OR interaction)))
AND
Concept 2:
TS=(Pediatrics OR Child OR children OR preschool OR preschoolers OR adolescent OR adolescents OR pediatric OR teen OR teens OR teenage OR "teen agers" OR teenagers OR youth OR youths)
NOT
Concept 3:

TS=("systematic review" OR "systematic literature review" OR "scoping review" OR "systematic narrative review" OR "systematic qualitative review" OR "systematic evidence review" OR "systematic quantitative review" OR "systematic meta-review" OR "systematic critical review" OR "systematic mixed studies review")
Concept 1 AND Concept 2 AND NOT Concept 3

Scopus Database
Concept 1:
TITLE-ABS-KEY ("Animal Assisted Therapy" OR "Therapy Animals" OR "Human-Animal Interaction")
OR TITLE-ABS (AAE OR "animal assisted" OR AAI OR AAT OR "Animal facilitated" OR "therapy animal" OR "therapy animals" OR "pet program" OR "pet programs" OR "pet support" OR "support animal" OR "support animals" OR "pet therapy" OR "pet assisted" OR "pet facilitated" OR "animal therapy" OR "animal program" OR "animal programs" OR "animal intervention" OR "animal interventions" OR "dolphin assisted" OR "human pet")
OR TITLE-ABS (hamster OR hamsters OR ferrets OR ferret OR "guinea pigs" OR "guinea pig" OR gerbil OR gerbils OR rabbits OR rabbit OR hares OR hare OR cats OR cat OR cow OR calf OR goat OR goats OR fish OR dolphin OR dolphins)
AND TITLE-ABS (facilitated OR therapy OR interaction)
AND
Concept 2:
TITLE-ABS-KEY ("Pediatrics" OR "Child" OR "Child, Preschool" OR "Adolescent")
OR TITLE-ABS ("Child" OR children OR preschool OR preschoolers OR adolescent OR adolescents OR pediatric OR pediatrics OR teen OR teens OR teenage OR "teen agers" OR teenagers OR youth OR youths)
AND NOT
Concept 3:
TITLE-ABS ("systematic review" OR "systematic literature review" OR "scoping review" OR "systematic narrative review" OR "systematic qualitative review" OR "systematic evidence review" OR "systematic quantitative review" OR "systematic meta-review" OR "systematic critical review" OR "systematic mixed studies review")
Concept 1 AND Concept 2 AND NOT Concept 3

CINAHL
Concept 1
S1:
MH "Animal Assisted Therapy" OR MH "Therapy Animals" OR MH "Human-Animal Interaction") OR TI (AAE OR "animal assisted" OR AAI OR AAT OR "Animal facilitated" OR "therapy animal" OR "therapy animals" OR "pet program" OR "pet programs" OR "pet support" OR "support animal" OR "support animals" OR "pet therapy" OR "pet assisted" OR "pet facilitated" OR "animal therapy" OR "animal program" OR "animal programs" OR "animal intervention" OR "animal interventions" OR "dolphin assisted" OR "human pet") OR AB (AAE OR "animal assisted" OR AAI OR AAT OR "Animal facilitated" OR "therapy animal" OR "therapy animals" OR "pet program" OR "pet programs" OR "pet support" OR "support animal" OR "support animals" OR "pet therapy" OR "pet assisted" OR "pet facilitated" OR "animal therapy" OR "animal program" OR "animal programs" OR "animal intervention" OR "animal interventions" OR "dolphin assisted" OR "human pet") OR ((TI (hamster OR hamsters OR ferrets OR ferret OR "guinea pigs" OR "guinea pig" OR gerbil OR gerbils OR rabbits OR rabbit OR hares OR hare OR cats OR cat OR cow OR calf OR goat OR goats OR fish OR dolphin OR dolphins) OR AB (hamster OR hamsters OR ferrets OR ferret OR "guinea pigs" OR "guinea pig" OR gerbil OR gerbils OR rabbits OR rabbit OR hares OR hare OR cats OR cat OR cow OR calf OR goat OR goats OR fish OR dolphin OR dolphins)) AND (TI (facilitated OR therapy OR interaction) OR AB (facilitated OR therapy OR interaction)

Concept 2
S2
MH "Pediatrics" OR MH "Child" OR MH "Child, Preschool" OR MH "Adolescent") OR TI ("Child" OR children OR preschool OR preschoolers OR adolescent OR adolescents OR pediatric OR pediatrics OR teen OR teens OR teenage OR "teen agers" OR teenagers OR youth OR youths) OR AB ("Child" OR children OR preschool OR preschoolers OR adolescent OR adolescents OR pediatric OR pediatrics OR teen OR teens OR teenage OR "teen agers" OR teenagers OR youth OR youths
Concept 3
S3
TI ("systematic review" OR "systematic literature review" OR "scoping review" OR "systematic narrative review" OR "systematic qualitative review" OR "systematic evidence review" OR "systematic quantitative review" OR "systematic meta-review" OR "systematic critical review" OR "systematic mixed studies review") OR AB ("systematic review" OR "systematic literature review" OR "scoping review" OR "systematic narrative review" OR "systematic qualitative review" OR "systematic evidence review" OR "systematic quantitative review" OR "systematic meta-review" OR "systematic critical review" OR "systematic mixed studies review")
S4 (S1 AND S2) NOT S3

PsycINFO
S1
DE "Animal Assisted Therapy" OR DE "Human Animal Interaction"
S2
TI (AAE OR AAI OR AAT OR "animal assisted" OR "animal facilitated" OR "therapy animal*" OR "pet program*" OR "pet support" OR "support animal*" OR "pet therapy" OR "pet assisted" OR "pet facilitated" OR "animal therapy" OR "animal program*" OR "animal intervention*" OR "dolphin assisted" OR "human pet") OR AB (AAE OR AAI OR AAT OR "animal assisted" OR "animal facilitated" OR "therapy animal*" OR "pet program*" OR "pet support" OR "support animal*" OR "pet therapy" OR "pet assisted" OR "pet facilitated" OR "animal therapy" OR "animal program*" OR "animal intervention*" OR "dolphin assisted" OR "human pet")
S3 TI (hamster* OR ferret* OR "guinea pig*" OR gerbil* OR rabbit* OR hare* OR cat* OR cow* OR calf OR calves OR goat* OR fish* OR dolphin*) OR AB (hamster* OR ferret* OR "guinea pig*" OR gerbil* OR rabbit* OR hare* OR cat* OR cow* OR calf OR calves OR goat* OR fish* OR dolphin*)
AND
TI (facilitated OR therapy OR interaction) OR AB (facilitated OR therapy OR interaction)
S4 S1 OR S2 OR S3
S5
DE "Pediatrics"
S6 TI (child* OR preschool* OR adolescen* OR pediatric* OR paediatric* OR teen* OR "teen ager*" OR youth*) OR AB (child* OR preschool* OR adolescen* OR pediatric* OR paediatric* OR teen* OR "teen ager*" OR youth*)
S7 S5 OR S6
S8
TI ("systematic review*" OR "systematic literature review*" OR "scoping review*" OR "systematic narrative review*" OR "systematic qualitative review*" OR "systematic evidence review*" OR "systematic quantitative review*" OR "systematic meta-review*" OR "systematic critical review*" OR "systematic mixed studies review*") OR AB ("systematic review*" OR "systematic literature review*" OR "scoping review*" OR "systematic narrative review*" OR "systematic qualitative

review*" OR "systematic evidence review*" OR "systematic quantitative review*" OR "systematic meta-review*" OR "systematic critical review*" OR "systematic mixed studies review*")

S9 S4 AND S7

S10 S9 NOT S8

Table S3: List of Excluded Studies at Full Text Screening Step and Reason for Exclusion

Study citation	Reason for exclusion
Anderson, H. K., Hayes, S. L., & Smith, J. P. (2019). Animal-assisted therapy in pediatric speech-language therapy with a preschool child with severe language delay: A single-subject design. *The Internet Journal of Allied Health Sciences and Practice*, 17(3), Article 1.	Wrong assisted animal species (domesticated dog or horse)
Antonioli, C., & Reveley, M. A. (2005). Randomised controlled trial of animal facilitated therapy with dolphins in the treatment of depression. *BMJ, 331*(7527), 1231.	Adult population
Balluerka, N., Muela, A., Amiano, N., & Caldentey, M. A. (2014). Influence of animal-assisted therapy (AAT) on the attachment representations of youth in residential care. *Children and Youth Services Review, 42,* 103–109.	Wrong assisted animal species (domesticated dog or horse)
Balluerka, N., Muela, A., Amiano, N., & Caldentey, M. A. (2015). Promoting psychosocial adaptation of youths in residential care through animal-assisted psychotherapy. *Child Abuse & Neglect, 50*, 193–205.	Wrong assisted animal species (domesticated dog or horse)
Banman, J. K. (1995). Animal-assisted therapy with adolescents in a psychiatric facility. *Journal of Pastoral Care, 49*(3), 274–278.	Wrong intervention
Barker, S. B., Knisely, J. S., Schubert, C. M., Green, J. D., & Ameringer, S. (2015). The effect of an animal-assisted intervention on anxiety and pain in hospitalized children. *Anthrozoös, 28*(1), 101–112.	Wrong assisted animal species (domesticated dog or horse)
Battaglini, C., Isaja, V., Riscossa, G., Giordano, M., Quarello, P., Zucchetti, G., & Fagioli, F. (2026). Animal-assisted psychoeducational intervention in paediatric oncohaematology: Evidence from a single-centre observational study. *Children, 13*(1), Article 136.	Remote interaction/no live animal
Borioni, N., Marinaro, P., Celestini, S., Del Sole, F., Magro, R., Zoppi, D., Mattei, F., Dall'Armi, V., Mazzarella, F., Cesario, A., & Bonassi, S. (2012). Effect of equestrian therapy and onotherapy in physical and psycho-social performances of adults with intellectual disability: A preliminary study of evaluation tools based on the ICF classification. *Disability and Rehabilitation, 34*(4), 279–287.	Adult population
Branson, S. M., Boss, L., Padhye, N. S., Trötscher, T., & Ward, A. (2017). Effects of animal-assisted activities on biobehavioral stress responses in hospitalized children: A randomized controlled study. *Journal of Pediatric Nursing, 36*, 84–91.	Wrong assisted animal species (domesticated dog or horse)
Braun, C., Stangler, T., Narveson, J., & Pettingell, S. (2009). Animal-assisted therapy as a pain relief intervention for children. *Complementary Therapies in Clinical Practice, 15*(2), 105–109.	Wrong assisted animal species (domesticated dog or horse)
Byström, K., Grahn, P., & Hägerhäll, C. (2019). Vitality from experiences in nature and contact with animals: A way to develop joint attention and social engagement in children with autism? *International Journal of Environmental Research and Public Health, 16*(23), Article 4673.	Wrong assisted animal species (domesticated dog or horse)
Chubak, J., & Hawkes, R. (2016). Animal-assisted activities: Results from a survey of top-ranked pediatric oncology hospitals. *Journal of Pediatric Oncology Nursing, 33*(4), 289–296.	Parent or therapist perspectives only
Chuprikov, A. P., Popovskiy, B. P., & Shypelik, T. V. (2013). Contemporary state of dolphin therapy. *Archiv Euromedica, 3*(1), 10–15.	Review/no original data

Every, D., Smith, K., Smith, B., Trigg, J., & Thompson, K. (2017). How can a donkey fly on the plane? The benefits and limits of animal therapy with refugees. *Clinical Psychologist*, 21(1), 44–53.	Parent or therapist perspectives only
Ferreira, A. O., Rodrigues, E. A. F., Santos, A. C., Guerra, R. R., Miglino, M. A., Maria, D. A., & Ambrósio, C. E. (2016). Animal-assisted therapy in early childhood schools in Sao Paulo, Brazil. *Pesquisa Veterinária Brasileira, 36*, 46–50.	Wrong outcomes
Flynn, E., Roguski, J., Wolf, J., Trujillo, K., Tedeschi, P., & Morris, K. N. (2019). A randomized controlled trial of animal-assisted therapy as an adjunct to intensive family preservation services. *Child Maltreatment, 24*(2), 161–168.	Wrong assisted animal species (domesticated dog or horse)
Fodstad, J. C., Bauers, J., Sexton, M., Butler, M., Karlsson, C., & Neff, M. (2019). Development of an animal-assisted activity program on a pediatric behavioral health unit. *Complementary Therapies in Clinical Practice, 36*, 153–157.	Wrong assisted animal species (domesticated dog or horse)
Guérin, N. A., Gabriels, R. L., Germone, M. M., Schuck, S. E. B., Traynor, A., Thomas, K. M., McKenzie, S. J., Slaughter, V., & O'Haire, M. E. (2018). Reliability and validity assessment of the Observation of Human–Animal Interaction for Research (OHAIRE) behavior coding tool. *Frontiers in Veterinary Science, 5*, Article 268.	Wrong outcomes
Guérin, N. A., Rodriguez, K. E., Brodhead, M. T., & O'Haire, M. E. (2017). Assessing preferences for animals in children with autism: A new use for video-based preference assessment. *Frontiers in Veterinary Science, 4*, Article 29.	Remote interaction/no live animal
Gupta, N., & Yadav, T. (2018). Parents' acceptance and their children's choice of pet for animal-assisted therapy (A.A.T.) in 3- to 12-year-old children in the dental operatory: A questionnaire-based pilot study. *International Journal of Paediatric Dentistry, 28*(4), 373–379.	Wrong intervention
Harada, T., Ishizaki, F., Cheng, W., Nitta, Y., Miki, Y., Numamoto, H., et al. (2020). Relationship between the characteristics of animal-assisted therapy and patients. *International Medical Journal*, 27(5), 620-623.	Wrong study design
Harkness, T., & Moorhead, A. (2025). Biopsychosocial benefits of animal-assisted activities for children aged 8–16 years from providers' perspectives. *International Journal of Adolescence and Youth*, 30(1).	Parent or therapist perspectives only
Hart, L. A., Thigpen, A. P., Willits, N. H., Lyons, L. A., Hertz-Picciotto, I., & Hart, B. L. (2018). Affectionate interactions of cats with children having autism spectrum disorder. *Frontiers in Veterinary Science, 5*, Article 39.	Relied on pet in home not animal-assisted therapy
Heimlich, K. (2001). Animal-assisted therapy and the severely disabled child: A quantitative study. *Journal of Rehabilitation, 67*(4), 48–54.	Wrong assisted animal species (domesticated dog or horse)
Hong, L., Shen, X., Hui, T. K., Luk, R. K., So, H. K., & Wong, W. H. (2025). Developing a measurement tool for assessing animal-assisted activity effectiveness on children with special educational needs' socialization: A pilot study. *SAGE Open Medicine, 13*, Article 20503121251363015.	Wrong assisted animal species (domesticated dog or horse)
Hyun, G. J., Jung, T. W., Park, J. H., Kang, K. D., Kim, S. M., Son, Y. D., Cheong, J. H., Kim, B. N., & Han, D. H. (2016). Changes in gait balance and brain connectivity in response to equine-assisted activity and training in children with attention deficit hyperactivity disorder. *Journal of Alternative and Complementary Medicine, 22*(4), 286–293.	Wrong assisted animal species (domesticated dog or horse)
Kaminski, M., Pellino, T., & Wish, J. (2002). Play and pets: The physical and emotional impact of child-life and pet therapy on hospitalized children. *Children's Health Care, 31*(4), 321–335.	Wrong assisted animal species (domesticated dog or horse)

Kapov, G., Linton, K., Gatewood, C., Liu, C., Strauman, T., Hodges, E., Graves, C., Sawicki, C., Wu, D., Divaris, K., & Jacox, L. A. (2025). Effects of animal-assisted therapy on dental anxiety, behavior, and perceptions in young pediatric patients: A blinded randomized controlled trial. *Trials, 26*(1), Article 279.	Wrong assisted animal species (domesticated dog or horse)
Koda, N., Kutsumi, S., Hirose, T., & Watanabe, G. (2016). Educational possibilities of keeping goats in elementary schools in Japan. *Frontiers in Veterinary Science, 3*, Article 118.	Wrong outcomes
Kohsaku, N., Cheng, W., Harada, T., Ishizaki, F., Nitta, Y., et al. (2020). Status and practice of therapy goat in the world. *International Medical Journal, 27*(5), 616–619.	Wrong outcomes
Kręgiel, A., Zaworski, K., & Kołodziej, E. (2019). Effects of animal-assisted therapy on parent-reported behaviour and motor activity of children with autism spectrum disorder. *Health Problems of Civilization, 13*(4), 273–278.	Parent or therapist perspectives only
Litta, A., Benazzi, D., Carbutti, P., Vacca, A., Nannavecchia, A. M., Morelli, A., Sisto, A. M., Attolino, E., Manigrasso, P., & Nacci, M. (2024). Animal-assisted intervention (AAI) in a recovery-oriented psychiatric rehabilitation program. *Psychiatria Danubina, 36*(Suppl. 2), 371–375.	Adult population
López-Fernández, E., Palacios-Cuesta, A., Rodríguez-Martínez, A., Olmedilla-Jodar, M., Fernández-Andrade, R., Mediavilla-Fernández, R., Sánchez-Díaz, J. I., & Máximo-Bocanegra, N. (2024). Implementation feasibility of animal-assisted therapy in a pediatric intensive care unit: Effectiveness on reduction of pain, fear, and anxiety. *European Journal of Pediatrics, 183*(2), 843–851.	Wrong assisted animal species (domesticated dog or horse)
Mezza, F., Saturnino, C., Pizzo, R., Santaniello, A., Cristiano, S., Garzillo, S., et al. (2022). Process evaluation of animal-assisted therapies with children: The role of the human-animal bond on the therapeutic alliance, depth of elaboration, and smoothness of sessions. *Mediterranean Journal of Clinical Psychology, 10*(2).	Wrong assisted animal species (domesticated dog or horse)
Moreno Escobar, J. J., Flores Hidalgo, B. L., Morales Matamoros, O., Coria Páez, A. L., & Quintana Espinosa, H. (2025). Electroencephalographic signal acquisition framework for neurodiverse: A case study of dolphin-assisted therapy. *Journal of Visualized Experiments*, (220).	Wrong study design
Muela, A., Azpiroz, J., Calzada, N., Soroa, G., & Aritzeta, A. (2019). Leaving a mark: An animal-assisted intervention programme for children who have been exposed to gender-based violence: A pilot study. *International Journal of Environmental Research and Public Health, 16*(21), Article 4084.	Wrong assisted animal species (domesticated dog or horse
Muela, A., Balluerka, N., Sansinenea, E., Machimbarrena, J. M., García-Ormaza, J., Ibarretxe, N., Eguren, A., & Baigorri, P. (2021). A social-emotional learning program for suicide prevention through animal-assisted intervention. *Animals, 11*(12), Article 3375.	Wrong assisted animal species (domesticated dog or horse)
Muela, A., Balluerka, N., Amiano, N., Caldentey, M. A., & Aliri, J. (2017). Animal-assisted psychotherapy for young people with behavioural problems in residential care. *Clinical Psychology & Psychotherapy, 24*(6), O1485–O1494.	Wrong assisted animal species (domesticated dog or horse)
Nalini, B., & Kumar, C. S. (2022). Animal-assisted therapy: A potential tool for management of dental anxiety in children: A questionnaire-based cross-sectional study. *Journal of Clinical and Diagnostic Research, 16*(2), ZC30–ZC34.	Wrong outcomes
Nammalwar, R. B., & Rangeeth, P. (2018). A bite out of anxiety: Evaluation of animal-assisted activity on anxiety in children attending a pediatric dental outpatient unit. *Journal of Indian Society of Pedodontics and Preventive Dentistry, 36*(2), 181–184.	Wrong assisted animal species (domesticated dog or horse)
Ngai, J. T. K., Yu, R. W. M., Chau, K. K. Y., & Wong, P. W. C. (2021). Effectiveness of a school-based programme of animal-assisted humane education in Hong Kong for the promotion of social and emotional learning: A quasi-experimental pilot study. *PLOS ONE, 16*(3), Article e0249033.	Wrong assisted animal species (domesticated dog or horse)

Potrich, T., Nitschke, R. G., Marques, M. I., & Viegas, S. M. (2021). Program of animal-assisted interventions for children with autism spectrum disorder. *Revista de Enfermagem Referência*, (7), 1–8.	Wrong assisted animal species (domesticated dog or horse)
Romanchuk, A. P., Beseda, V. V., Podgornaya, V. V., & Sorokin, M. Y. (2012). Effect of dolphin therapy on the muscle tone of children suffering autism. *Science and Education*, (2).	Unable to acquire publication
Schuck, S. E. B., Johnson, H. L., Abdullah, M. M., Stehli, A., Fine, A. H., & Lakes, K. D. (2018). The role of animal-assisted intervention on improving self-esteem in children with attention-deficit/hyperactivity disorder. *Frontiers in Pediatrics, 6*, Article 300.	Wrong assisted animal species (domesticated dog or horse)
Shenk, C. E., Allen, B., Dreschel, N. A., Wang, M., Felt, J. M., Brown, M. P., Bucher, A. M., Chen, M. J., & Olson, A. E. (2022). Respiratory sinus arrhythmia change during trauma-focused cognitive-behavioral therapy: Results from a randomized controlled feasibility trial. *Research on Child and Adolescent Psychopathology, 50*(11), 1487–1499.	Wrong assisted animal species (domesticated dog or horse)
Solé, M., Camerino, O., Rodrigo, M., Jonsson, G., Prat, Q., & Castañer, M. (2023). Patterns of interactive and motor behavior: Animal-assisted intervention in inclusive education. *Apunts Educación Física y Deportes, 151*, 17–26.	Wrong assisted animal species (domesticated dog or horse)
Stefanini, M. C., Martino, A., Allori, P., Galeotti, F., & Tani, F. (2015). The use of animal-assisted therapy in adolescents with acute mental disorders: A randomized controlled study. *Complementary Therapies in Clinical Practice, 21*(1), 42–46.	Wrong assisted animal species (domesticated dog or horse)
Stefanini, M. C., Martino, A., Bacci, B., & Tani, F. (2016). The effect of animal-assisted therapy on emotional and behavioral symptoms in children and adolescents hospitalized for acute mental disorders. *European Journal of Integrative Medicine, 8*(2), 81–88.	Wrong assisted animal species (domesticated dog or horse)
Stoffel, J. M., & Braun, C. A. (2006). Animal-assisted therapy: An analysis of patient testimonials. *Journal of Undergraduate Nursing Scholarship, 8*(1), 6.	Wrong assisted animal species (domesticated dog or horse)
Suba-Bokodi, É., Nagy, I., & Molnár, M. (2022). Changes in the stress tolerance of dwarf rabbits in animal-assisted interventions. *Applied Sciences, 12*(14), Article 6979.	Wrong outcomes
Topel, E.-M., & Lachmann, F. M. (2008). Life begins on an ant farm for two patients with Asperger's syndrome. *Psychoanalytic Psychology, 25*(4), 602–617.	Wrong study design
Topel, E. M., & Lachmann, F. M. (2011). Connecting with two Asperger's syndrome patients—with the help of some ants. *Psychoanalytic Inquiry, 31*(3), 303–319.	Wrong outcomes
Trujillo, K. C., Kuo, G. T., Hull, M. L., Ingram, A. E., & Thurstone, C. C. (2020). Engaging adolescents: Animal-assisted therapy for adolescents with psychiatric and substance use disorders. *Journal of Child and Family Studies, 29*(2), 307–314.	Wrong assisted animal species (domesticated dog or horse)
Tsai, C. C., Friedmann, E., & Thomas, S. A. (2010). The effect of animal-assisted therapy on stress responses in hospitalized children. *Anthrozoös, 23*(3), 245–258.	Wrong assisted animal species (domesticated dog or horse)
Wagner, C., Lang, U. E., & Hediger, K. (2019). "There is a cat on our ward": Inpatient and staff member attitudes toward and experiences with cats in a psychiatric ward. *International Journal of Environmental Research and Public Health, 16*(17), Article 3108.	Adult population
Wong, P. W., Yu, R. W., Li, T. M., Lai, S. L., Ng, H. Y., & Fan, W. T. (2019). Efficacy of a multi-component intervention with animal-assisted therapy for socially withdrawn youth in Hong Kong. *Society & Animals, 27*(5–6), 614–627.	Wrong assisted animal species (domesticated dog or horse)

Wu, A. S., Niedra, R., Pendergast, L., & McCrindle, B. W. (2002). Acceptability and impact of pet visitation on a pediatric cardiology inpatient unit. *Journal of Pediatric Nursing, 17*(5), 354–362.	Wrong assisted animal species (domesticated dog or horse)
Yelvington, M., Spray, B., Bregy, F., Ramick, A., Lovenstein, A., & Walden, M. (2025). Impact of animal-assisted intervention on therapy engagement in hospitalized children with traumatic brain injuries. *PM&R, 17*(7), 804–810.	Wrong assisted animal species (domesticated dog or horse)
Yurdadön, N., Bertan, İ., & Şahin, R. S. (2025). The effects of animal-assisted therapy on adolescent and young adults with intellectual disabilities: A qualitative study based on caregivers' perspectives. *Scandinavian Journal of Caring Sciences, 39*(3), e70117.	Parent or therapist perspectives only
Žalienė, L., Mockevičienė, D., Kreivinienė, B., Razbadauskas, A., Kleiva, Ž., & Kirkutis, A. (2018). Short-term and long-term effects of riding for children with cerebral palsy gross motor functions. *BioMed Research International, 2018*, Article 4190249.	Wrong assisted animal species (domesticated dog or horse)

Table S4. Animal Species Included in Pediatric Non-Traditional Animal-Assisted Intervention Studies and Characteristics of Their Use

**Study ID**	**Common name**[1]	**Scientific name**	**Single- vs mixed-species intervention**	**Comments**
Boyer & Mundschenk (2014)	Cat	*Felis catus*	Single-species	Live cat condition compared with toy cat and preferred activity.
Breitenbach et al. (2009)	Dolphin; farm animals in comparison group	Dolphin species not reported; farm-animal species not reported	Mixed across study arms	DAT arm single-species at category level; farm-animal comparison group not species-specific.
Byström et al. (2022)	Farm animals	Not reported	Mixed-species	Species not identified in available text.
Conniff et al. (2005)	Dog, cat, rabbit, llama	*Canis familiaris; Felis catus; Oryctolagus cuniculus; Lama glama*	Mixed-species	Pet-visitation program.
Corallo et al. (2023)	Donkey	*Equus africanus asinus*	Single-species	Onotherapy/donkey-assisted therapy.
De Rose et al. (2011)	Donkey	*Equus africanus asinus*	Single-species	Donkey-assisted rehabilitation.
Demiralay & Keser (2022)	Persian Chinchilla cat	*Felis catus*	Single-species	Breed/type reported; domestic cat scientific name used.
Dicé at al. (2017)	Dog; donkey	*Canis familiaris; Equus africanus asinus*	Mixed-species	AAE intervention included one dog and three donkeys in a primary school setting.
Dilts et al. (2011)	Dolphin	Not reported	Single-species	Dolphin species not identified in available text.
Dolecheck et al. (2024)	Horse, miniature horse, goat, rabbit, cat, dog	*Equus caballus; Capra hircus; Oryctolagus cuniculus; Felis catus; Canis familiaris*	Mixed-species	Farm-based AAT program using multiple animal species within the Horse Boy Method framework.

Study ID	Common name[1]	Scientific name	Single- vs mixed-species intervention	Comments
Flynn et al. (2024)	Farm animals, equines, dogs, wildlife animals	Multiple species not fully specified	Mixed-species	Green Chimneys program incorporated farm animals, equine, canine, and wildlife programs. Species not consistently specified.
Hirschenhauser & Brodesser (2023)	Giant African land snail	*Achatina fulica*	Single-species	Classroom-based educational study involving one Giant African land snail.
Geldhof et al. (2021)	Farm and wildlife animals	Not reported	Mixed-species	Species not identified in available text.
Griffioen & Enders-Slegers (2014)	Dolphin	Not reported	Single-species	Dolphin species not identified in available text.
Griffioen et al. (2019)	Dolphin	Not reported	Single-species	Dolphin species not identified in available text.
Harada et al. (2019)	Tokara goat	*Capra aegagrus f. hircus*	Single-species	Therapy goat described as Tokara-goat lineage.
Hernandez-Espeso et al. (2021)	Bottlenose dolphin	*Tursiops truncatus*	Single-species	Species explicitly reported.
Ivancsik et al. (2025)	Lionhead dwarf rabbit	*Oryctolagus cuniculus*	Single-species	Specially trained therapy rabbit in classroom.
Kim (2024)	Rabbit	*Oryctolagus cuniculus*	Single-species	One-year-old rabbits used as helper animals.
Kim et al. (2018)	Cabbage white butterfly, swallowtail butterfly, stag beetle, rhinoceros beetle, silkworm, oriental garden cricket	*Pieris rapae*; swallowtail species not reported; stag beetle species not reported; rhinoceros beetle species not reported; *Bombyx mori; Teleogryllus sp*. (species not confirmed)	Mixed-species	Children selected three insect types from six options; several insects not identified to species in article text available.
Kreiviniene & Aciene (2026)	Dolphin	Not reported	Single-species	Species not reported; policy/interview study.

Study ID	Common name[1]	Scientific name	Single- vs mixed-species intervention	Comments
Kreiviniene & Kleiva (2017)	Dolphin	Not reported	Single-species	Dolphin species not identified in available text.
Locher et al. (2023)	Horse, rabbit, chicken, goat, dog	*Equus caballus; Oryctolagus cuniculus; Gallus gallus domesticus; Capra hircus; Canis familiaris*	Mixed-species	Animal-assisted group psychotherapy used multiple species.
Matamoros et al. (2020)	Dolphin	Not reported	Single-species	DAT neurodynamics study; species not reported in available text.
Matamoros et al. (2022)	Bottlenose dolphin	*Tursiops truncatus*	Single-species	Female bottlenose dolphin explicitly reported.
Molnar et al. (2020)	Dwarf rabbit	*Oryctolagus cuniculus*	Single-species	Dwarf rabbits selected for classroom intervention.
Moreno Escobar et al. (2023)	Dolphin	Not reported	Single-species	Dolphin species not reported in available text; study focuses on emotion-detection methods during DAT.
Moreno Escobar et al. (2024)	Bottlenose dolphin	*Tursiops truncatus*	Single-species	DAT biomarker study involving dolphin-assisted therapy for pediatric cerebral palsy.
Mudaly et al. (2014)	Guinea pig, rabbit, rat	*Cavia porcellus; Oryctolagus cuniculus; Rattus norvegicus*	Mixed-species	Small animals chosen for the AAET group.
Murry & Allen (2012)	Bearded dragon	*Pogona sp*. (likely *Pogona vitticeps;* species not confirmed)	Single-species	Participants selected bearded dragons; article did not identify species to Latin binomial in available text.
Nathanson et al (2007)	Atlantic bottlenose dolphin	*Tursiops truncatus*	Single-species	

Study ID	Common name[1]	Scientific name	Single- vs mixed-species intervention	Comments
Novotna et al. (2025)	Horse, sheep, goat, pig, rabbit, chicken	*Equus caballus; Ovis aries; Capra hircus; Sus scrofa domesticus; Oryctolagus cuniculus; Gallus gallus domesticus*	Mixed-species	Farm animals explicitly listed in outdoor lesson.
O'Haire et al. (2013a)	Guinea pig	*Cavia porcellus*	Single-species	Two guinea pigs used in animal condition.
O'Haire et al. (2013b)	Guinea pig	*Cavia porcellus*	Single-species	Classroom AAA program used pairs of guinea pigs.
O'Haire et al. (2014)	Guinea pig	*Cavia porcellus*	Single-species	Pairs of guinea pigs housed in classrooms for AAA program.
O'Haire et al. (2015)	Guinea pig	*Cavia porcellus*	Single-species	Social-buffer physiology study used guinea pigs in the animal free-play condition.
Salgueiro et al. (2012)	Dolphin	Not reported	Single-species	Dolphin interaction program; species not reported in extracted text.
Sarman & Günay (2023)	Goldfish	*Carassius auratus*	Single-species	Hospitalized children cared for/observed goldfish.
Sarman & Tuncay (2024)	Goldfish and aquatic turtle	*Carassius auratus*; aquatic turtle species not reported	Mixed-species across intervention arms	Three-arm RCT comparing fish, turtle, and control; turtle species not reported.
Satiansukpong et al. (2016)	Thai elephant / Asian elephant	*Elephas maximus*	Single-species	Thai Elephant-Assisted Therapy Programme.
Sokal & Kahl (2019)	Rabbit and dog	*Oryctolagus cuniculus; Canis familiaris*	Mixed-species across program arms	Effects reported for rabbit club and dog club; species-specific findings should be coded separately if possible.

Study ID	Common name[1]	Scientific name	Single- vs mixed-species intervention	Comments
Stumpf & Breitenbach (2014)	Dolphin	Not reported	Single-species at intervention level	Four dolphins were present in the pool; species not reported in extracted text.
Tahan et al. (2022)	Animals (species not reported)	Not reported	Unclear	Full text excerpt does not identify the species used in AAT; code as not reported until audited.
Torquati et al. (2025)	Red Pied Valdostana cow / domestic cattle	*Bos taurus*	Single-species	Four Red Pied Valdostana cows.
Wedl & Kotrschal (2009)	Rabbit	*Oryctolagus cuniculus*	Single-species	Several rabbits were present, but all were the same species.
Yilmaz & Demir Acar (2024)	Goldfish	*Carassius auratus*	Single-species	Species common name reported; scientific name coded as standard goldfish.
MdYusof & Chia (2012)	Indo-Pacific humpback dolphin	*Sousa chinensis*	Single-species	Species explicitly reported.
Zagrai et al. (2024)	Netherland Dwarf rabbit; rabbit	*Oryctolagus cuniculus*	Single-species	Breed reported as Netherland Dwarf; intervention also assessed rabbit stress/welfare.

[1]Unless otherwise noted species are considered domesticated (e.g., cat, dog, donkey, rabbit)

Table S5. Characteristics of Included Non-Traditional Animal-Assisted Intervention Studies Organized by Animal Species and Intervention Type

Study ID	Country	Aims	Participants	Study design / data collection	Key findings[1]
Dolphin/Intervention Studies					
Breitenbach et al. (2009)	Germany	To test whether a DAT program improved communication and social-emotional functioning in children with severe disabilities, and to separate effects of dolphin interaction from parent consultation and environment. Study attempted to distinguish dolphin interaction effects; parent consultation effects; and environmental effects,	118 children aged 5-8 years with severe communication limitations associated with autism, Down syndrome, mental disability, or physical disability; four groups: experimental DAT n=40, non-treatment n=29, outpatient dolphin-only n=25, farm-animal comparison n=24.	Controlled before-after comparison with three control/comparison groups. Measures at baseline, 4 weeks post-treatment, and 6 months post-treatment included parent and professional questionnaires and videotaped parent-child interactions.	Parent questionnaires indicated short- and longer-term improvements in the full DAT group in verbal speech, non-verbal reactivity, social-emotional competence, and self-confidence. Dolphin-only sessions improved communication outcomes but not broader social-emotional outcomes. Professional ratings did not generally confirm parent-reported effects. Farm-animal comparison did not show the same parent-reported treatment effects.
Dilts et al. (2011)	Ukraine	To evaluate behavioral change in children with special needs participating in the multimodal DolphinSwim DAT program using a standardized parent-report measure.	37 parents of children with special needs enrolled in five DolphinSwim summer programs; diagnoses included cerebral palsy, developmental delay/disorder, intellectual disability, autism, epilepsy, Rett syndrome, Down syndrome, and others.	Convenience-sample pretest-posttest pilot without control group. Parents completed the Behavior Dimensions Rating Scale before and after a 2-week multimodal program that included multimodal dolphin sessions plus cognitive therapy, physiotherapy, cranio-sacral therapy, art therapy, psychotherapy, and alpha-sphere sessions.	Among participants with complete total data, parent-reported total behavior scores improved significantly. After adjustment, inattentive/irresponsible, socially withdrawn, and fearful/anxious subscales improved; aggressive/acting-out did not. Multimodal design limits its attribution to dolphins.

Study ID	Country	Aims	Participants	Study design / data collection	Key findings[1]
Griffioen & Enders-Slegers (2014)	Nether-lands	To examine whether DAT improved cognitive/social functioning in children with Down syndrome, especially verbalization, task performance, and social behavior.	45 children with Down syndrome aged 6-10 years with IQ > 40; three groups with DAT, swimming-pool control period, or waiting-list control period before DAT.	Prospective semi-crossover design. DAT consisted of weekly one-hour sessions for 6 weeks. Matson Evaluation of Social Skills for Individuals with Severe Retardation measured verbalization, impulsiveness, rule understanding, recognition of persons, and establishing contacts before/after DAT and control periods, with 6-month follow-up.	No significant changes occurred during swimming-pool or waiting-list control periods. After DAT, verbalization and recognition of persons improved significantly and impulsiveness decreased; rule understanding and establishing contacts did not significantly change. Verbalization continued to increase at follow-up.
Griffioen et al. (2019)	Nether-lands	To characterize verbal interactional synchrony/turn-taking between therapists and children with ASD during DAT and examine whether synchrony changed across six sessions.	Five children with ASD, 4 male and 1 female, aged 6-8.5 years, recruited through the SAM Foundation; three used single words and two used short sentences.	Five-case observational study. Children received six DAT sessions at Dolphinarium Harderwijk. Videos of first and last sessions were coded for therapist-child verbal turn-taking and analyzed with cross-recurrence quantification analysis.	Synchrony was detected in all therapist-child dyads. Adequate turn-taking appeared to increase over therapy but improvements varied and were greatest among children with better baseline verbal communication.
Hernandez-Espeso et al. (2021)	Spain	To test whether DAT improved social and communicative skills in children with ASD compared with the same therapy delivered without dolphins.	48 children aged 4-5 years with ASD and clear communication impairment; final DAT n=24 and therapy-without-dolphins n=19 after drop-outs.	Randomized pretest-posttest controlled design lasting 10 weeks. Intervention phase was 6 weeks with three 45-minute sessions per week. Both groups received the same therapeutic context except dolphin interaction. Measures included ADOS-G, Reynell Developmental Language Scales, and Vineland Adaptive Behavior Scales.	Both groups improved on most total scale scores. DAT showed significantly greater improvement only for two ADOS-G language/communication items: frequency of vocalizations toward others and gestures. Most gains were not dolphin-specific.

Study ID	Country	Aims	Participants	Study design / data collection	Key findings[1]
Kreiviniene & Kleiva (2017)	Lithuania	To describe subjective welfare understanding of families and dolphins in DAT and evaluate family emotional responses and perceived outcomes.	Eight families raising children with disabilities: one child with cerebral palsy, two with Down syndrome, and five with ASD.	Pilot mixed-methods/triangulation study during a 2-week holistic DAT program. Methods included eye tracking, facial-expression recognition, observational event logging, pre-program qualitative telephone interviews, and one-month post-program open-response evaluation.	Families were mostly happy while observing their children. Families of children with severe disability were most satisfied. Parents perceived dolphin welfare as involving the dolphin's ability to choose a child for communication. Measurable therapy goals were associated with more satisfying parent-reported outcomes one month later.
MdYusof & Chia (2012)	Singapore	To evaluate the 12-month Dolphin Encounter for Special Children (DESC) program for children with autism, focusing on stereotyped behavior, communication, social interaction, and Autism Quotient scores.	15 children with high-functioning autism, 10 boys and 5 girls, aged 9-10 years at program start.	Quasi-experimental pre/post design without a control group. Outcomes were measured before and after the DESC program with the Gilliam Autism Rating Scale.	Reported significant reductions in stereotyped behavior and significant improvements in communication, social interaction, and Autism Quotient with large effect sizes. Lack of control group limits attribution to DAT.
Salgueiro et al. (2012)	Portugal	To explore developmental and behavioral effects of a dolphin interaction program in children with ASD.	10 children diagnosed with ASD participated in a dolphin interaction program.	Exploratory pre/post evaluation using Childhood Autism Rating Scale, Psychoeducational Profile-Revised, Autism Treatment Evaluation Checklist, Theory of Mind tasks, and a custom Interaction Evaluation Grid for in-pool behavior complexity.	CARS, ATEC, and Theory of Mind tasks did not show benefits. PEP-R suggested statistically significant effects on overall development, fine motor development, cognitive performance, and cognitive verbal development. The Interaction Evaluation Grid showed significant evolution in behavioral complexity. Authors concluded that the study did

Study ID	Country	Aims	Participants	Study design / data collection	Key findings[1]
					not support significant developmental progress from the program.
Stumpf & Breitenbach (2014)	Germany	To evaluate a family-centered DAT program with purposeful parental involvement for children with severe disabilities.	47 children aged 5-10 years with severe communication limitations and disabilities including Down syndrome, physical disability, or mental retardation, plus parents and pedagogic/therapeutic staff.	Controlled pretest-posttest design with matched samples. Experimental families completed a 10-day DAT program at Tiergarten Nuremberg; controls were non-treatment matched children. Assessments occurred 4 weeks before therapy, 4 weeks after, and 6 months after. Parent and staff questionnaires assessed communication, social-emotional behavior, and parental quality of life.	Parent questionnaires showed stable positive changes in child communicative abilities, child social-emotional behavior, and parental quality of life, often with large effect sizes. Staff questionnaires showed only moderate confirmation for one communication factor and did not confirm social-emotional gains.
Dolphin/Mechanistic Studies					
Matamoros et al. (2020)	Mexico	To develop and test a brain-computer/EEG system for analyzing neurodynamics of children during DAT using power spectrum density and self-affine/fractal analysis.	Five child participants across two experiments: three children with infantile spastic cerebral palsy, one child with obsessive compulsive disorder, and one neurotypic child, studied at Definiti Ixtapa-Mexico facilities.	Physiological measurement study using TGAM1/NeuroSky EEG biosensor. EEG raw data were collected before and during DATfor Experiment 1 and before, during, and DAT for Experiment 2. Analyses used power spectrum density and self-affine analysis.	Power spectrum results showed a large increase in brain activity during DAT relative to before/after periods, reported around 376.28%. Self-affine analysis indicated anti-persistent behavior across children. Authors proposed longer DAT sampling windows and stationarity criteria as potential indicators of therapy efficiency.
Matamoros et al. (2022)	Mexico	To explore EEG signals from a patient with infantile spastic cerebral palsy undergoing DAT and	Single case study of one child with infantile spastic cerebral palsy; DAT session involved a therapist,	Four-stage cybernetic/health-informatics model: signal acquisition, EEG processing, EEG exploring, and	Power spectra of EEG and dolphin bioacoustic signals showed similar density patterns during DAT and

Study ID	Country	Aims	Participants	Study design / data collection	Key findings[1]
		bioacoustic signals emitted by a female bottlenose dolphin using nonlinear mathematical tools.	dolphin trainer, patient, and a female bottlenose dolphin.	HIS-DAT. EEG and hydrophone/bioacoustic signals were collected before, during, and after DAT and analyzed using power spectrum density and self-affine analysis.	the child's brain activity increased to approximately threefold higher-frequency activity when the therapist-dolphin pair interacted with the patient. Self-affine analysis suggested negative correlations in the patient's brain activity, with greatest changes during DAT.
Moreno Escobar et al. (2023)	Mexico	To develop and test deep convolutional neural network methods for detecting facial emotions in children with Down syndrome during DAT, and to explore whether adding EEG information improved emotion classification.	Image-based methodological study using 20,214 images in an exploratory database and 1,445 images in a Down Syndrome Dataset for training, validation, and testing. Focus population was a child with Down syndrome during DAT.	Computational/measurement study. Two Convolutional Neural Network (CNN) architectures were trained and evaluated; facial-emotion images were analyzed alone and in combination with EEG activity. The paper also discusses brain activity during DAT as contextual evidence.	Convolutional Neural Network (CNN) accuracy was 79% on a large reference database. When trained/tested on the Down Syndrome Dataset, accuracy was 72%; including EEG features increased average precision to 81%. Authors framed this as a tool for monitoring engagement and emotion during DAT, not as direct evidence of efficacy.
Moreno Escobar et al. (2024)	Mexico	To evaluate the use of a Siamese neural network as an objective biomarker for assessing the effects of DAT in children with spastic cerebral palsy.	Four children with spastic cerebral palsy undergoing DAT.	Mechanistic/physiological study using EEG, event-related potential (ERP), fast Fourier transform (FFT), and Siamese neural network analyses before and after DAT.	The model identified distinct EEG patterns associated with DAT, suggesting potential utility as an objective biomarker for monitoring treatment response. Findings indicate DAT may activate specific brain regions during intervention.
Dolphin/Experimental Studies					

Study ID	Country	Aims	Participants	Study design / data collection	Key findings[1]
Nathanson (2007)	USA	To compare reinforcement effectiveness of real Atlantic bottlenose dolphins and an animatronic dolphin (TAD) for children with disabilities.	35 children with disabilities representing 10 diagnoses from 7 countries.	ABA experimental comparison study measuring orienting responses and response times	No significant differences between real dolphins and TAD for most orienting responses. TAD was as effective or more effective than dolphins as a reinforcer, especially for some profoundly disabled children.
Dolphin/Implementation and Perception Studies					
Kreiviniene & Aciene (2026)	Lithuania	To examine links between conventional medicine and complementary/alternative medicine through parent perceptions of DAT as a health innovation in Lithuania.	147 interviews with parents whose children with disabilities participated in DAT.	Qualitative content-analysis study situated in health policy/legal-regulation context. Interviews addressed parental views of DAT, CAM/conventional medicine integration, and health-system innovation.	Parents viewed DAT as a complex, integrated program closer to conventional medicine than CAM because it combined multiple methods and professional supports. The paper emphasizes Lithuanian regulatory developments and opportunities for integrative medicine.
Rabbits and Guinea Pigs/Intervention Studies					
Ivancsik et al. (2025)	Hungary	To test whether rabbit-assisted intervention could support the preschool-to-school transition by reducing anxiety in first-grade children. Focus on animal-assisted pedagogy.	16 first-grade children in a Hungarian primary school.	One-group pretest-posttest study over 24 weeks with alternating 6-week periods without and with a trained therapy rabbit in the classroom. State and trait anxiety were measured repeatedly.	State and trait anxiety decreased more during rabbit-assisted periods than during periods without the animal. Effects were strongest among children with higher initial anxiety. Authors concluded rabbit-assisted pedagogy may support school transition.
Kim (2024)	Republic of Korea	To evaluate whether rabbit-based animal-assisted activities improve self-esteem in preschool children.	20 six-year-old children from two daycare centers, randomly assigned to animal assisted activities with rabbits n=10 or non- animal	Controlled pre/post activity study. The animal assisted activities group received six one-hour sessions twice weekly with two helper	Rabbit-based activities were associated with statistically significant increases in some self-esteem areas; effects were more evident among

Study ID	Country	Aims	Participants	Study design / data collection	Key findings[1]
			assisted activities comparison using videos/pictures n=10. Each group had 5 boys and 5 girls.	rabbits, one host, two assistant hosts, an animal manager, and an observer. Self-esteem was measured before and after the program.	boys than girls. Some self-esteem subareas decreased, so the pattern was not uniformly positive.
Molnar et al. (2020)	Hungary	To analyze effects of rabbit-assisted intervention on anxiety levels of first-grade children in classroom settings and compare effects across classes with different educational backgrounds.	27 first-grade children from two elementary school classrooms, including one integrating classroom with a high proportion of students with special educational needs.	Repeated-measures classroom study comparing alternating six-week periods with and without rabbit-assisted interventions. Anxiety was assessed using the State–Trait Anxiety Inventory for Children (STAI-C).	Rabbit-assisted interventions significantly reduced anxiety levels among first-grade students. Effects were strongest among children with higher baseline anxiety and in integrating classrooms serving students with special educational needs. Rabbits displayed behaviors consistent with positive welfare and voluntarily interacted with children.
O'Haire et al. (2013a)	Australia	To examine whether children with ASD show different social behavior with an adult and typically developing peers in the presence of animals compared with toys.	99 children from 15 classrooms in 4 schools; final groups comprised 33 children with ASD and 66 typically developing peers, arranged in triads.	Blinded observational comparison. Each triad completed three 10-minute free-play sessions with toys and three 10-minute free-play sessions with two guinea pigs. Video data were coded by blinded observers and analyzed with hierarchical generalized linear modeling.	Children with ASD showed more social approach behaviors, received more social approaches from peers, and displayed more prosocial behavior and positive affect in animal sessions than toy sessions. They showed less self-focused behavior and negative affect in animal sessions.
O'Haire et al. (2013b)	Australia	To test whether classroom-based animal-assisted activities with guinea pigs improved social functioning in primary school children.	128 primary school children aged 4.8-12.7 years; experimental animal assisted activities group n=64 and waitlist control group n=64.	Controlled waitlist study. Parents and teachers completed the Social Skills Rating System before and after an 8-week period. Teachers also rated academic competence.	Children in the guinea-pig animal assisted activities program showed significantly greater improvements than controls in teacher-rated social skills and parent/teacher-rated

Study ID	Country	Aims	Participants	Study design / data collection	Key findings[1]
					problem behaviors. Academic competence did not differ. The authors concluded that guinea-pig animal assisted activities may be feasible for improving classroom social functioning.
O'Haire et al. (2014)	Australia	To implement and evaluate a classroom-based animal-assisted activities program on social functioning in children with ASD.	64 children with ASD aged 5-12 years across 41 classrooms in 15 schools; 37 were in an 8-week waitlist group and 27 in a non-waitlist group.	Multisite control-to-intervention design. The animal assisted activities program included 8 weeks of guinea-pig exposure in the classroom plus sixteen 20-minute animal-interaction sessions. Teacher and parent standardized measures were collected at study entry, after waitlist/pre-intervention, and post-intervention.	Social functioning improved after the animal assisted activities program but not during the waitlist period: social approach behaviors and social skills increased, and social withdrawal decreased. More than half of parents reported increased child interest in attending school during the program.
Zagrai et al. (2024)	Romania	To assess whether rabbit-assisted therapy-related activities produced measurable stress responses in therapy rabbits and to evaluate effects of an existing rabbit-assisted therapy program on social communication, executive function, and motor-related behaviors in children with ASD. Study assessed both child outcomes and rabbit welfare.	10 children (6 boys and 4 girls) aged 7-13 years with ASD recruited from the School Centre for Inclusive Education LACRIMA Bistrița; 16 clinically healthy Netherland Dwarf rabbits (10 females, 6 castrated males), mostly aged 5-7 years.	Pilot pre/post field study of an existing rabbit-assisted therapy program. Rabbit stress was assessed with hematologic indicators, including hematocrit/polycythemia patterns. Child outcomes were summarized with behavioral indicators including hyperactivity, inappropriate speech, stereotypies, compliance, attention in activity, and communication. Descriptive statistics and Student t-tests were used.	Rabbit hematologic changes suggested transient polycythemia that diminished by the end of the experiment, interpreted by the authors as possible eustress rather than sustained distress. Child outcomes improved across several domains: hyperactivity decreased 31.5%, inappropriate speech 21.42%, stereotypies 66.6%, compliance improved 57.80%, attention in activity improved 30.30%, and

Study ID	Country	Aims	Participants	Study design / data collection	Key findings[1]
					communication showed the largest reported difference (93.75%).
Rabbits and Guinea Pigs/Mechanistic Studies					
O'Haire et al. (2015)	Australia/USA	To test whether animals function as social buffers by reducing physiological arousal in children with ASD during social interaction.	Children with ASD and typically developing peers in a school/social context; 99 children (33 with ASD and 66 typically developing peers), likely drawn from the O'Haire guinea-pig classroom/social-context study sample (O'Haire et al. (2013b).	Experimental social-context physiology study. Skin conductance arousal was measured continuously across four conditions: silent reading baseline, scripted reading-aloud classroom activity, free play with peers and toys, and free play with peers and guinea pigs.	Children with ASD showed higher arousal than typically developing children in all conditions except when animals were present. In the animal condition, children with ASD showed a 43% decrease in skin conductance responses during free play with peers compared with toys, supporting an anxiolytic/social-buffer interpretation.
Rabbits and Guinea Pigs/Human–Animal Relationship and Observational Studies					
Wedl & Kotrschal (2009)	Austria	To examine how preschool children's interest in animal contact was related to social competence, personality, age, gender, sibling status, and pet ownership.	50 preschool children aged 3-7 years (28 girls, 22 boys) at a preschool in Krems, Austria, with free access to rabbits during the observation period.	Naturalistic observational study. Video and direct observations captured frequency and duration of children's direct occupation, indirect occupation, and proximity to rabbits. Teachers rated personality/social status; data were analyzed with generalized linear models.	Girls, children with siblings, and children without pets were more oriented toward rabbits. Older children engaged less frequently but for longer when they did engage. Children rated as more confident/respected and less patient/calm, cheerful/sociable, and solitary spent more time directly occupied with rabbits. Results supported the interpretation that socially competent children were particularly interested in animals.
Cats/Intervention Studies					

Study ID	Country	Aims	Participants	Study design / data collection	Key findings[1]
Boyer & Mundschenk (2014)	USA	To determine whether a live cat promoted social communication between children with language impairments and typically developing peers compared with a toy cat and preferred play activities.	Three girls aged 4 years 4 months to 8 years 5 months with language impairments and documented peer-interaction difficulties; each paired with a typically developing peer.	Single-subject alternating-treatment design over 12 weeks. Dyads received 15-minute speech-language therapy sessions alternating live cat, toy cat, and preferred activity conditions; sessions were videotaped and coded; teacher social-skill ratings were collected.	Two of three participants demonstrated more sustained peer interaction and verbal communication in the live-cat condition than in the toy-cat or preferred-activity conditions. Teacher ratings suggested limited classroom generalization for some participants. Effects were variable and limited by sample size and attendance.
Demiralay & Keser (2022)	Türkiye	To determine the effect of pet (cat) therapy on stress and social anxiety levels among physically disabled children.	44 physically disabled children aged 8-11 years from two special education and rehabilitation centers; intervention n=21, control n=23.	Single-blind randomized controlled pre-test/post-test/follow-up study. Intervention group received a 7-week pet-therapy program with a four-year-old Persian Chinchilla cat; controls received routine center education. Outcomes included Perceived Stress Scale, Social Anxiety in Children Scale-Revised, and blood pressure measures.	Perceived stress and social anxiety decreased significantly in the pet-therapy group compared with controls after intervention and remained lower at follow-up relative to baseline. Blood pressure findings were mixed.
Farm Animals and Livestock/Donkeys/Intervention Studies					
Corallo et al. (2023)	Italy	To test whether donkey-assisted therapy added to neuropsychological group therapy improved self-esteem and school performance in children with specific learning disorders/dyslexia.	16 children with dyslexia, 8 male and 8 female, aged 7-12 years; experimental donkey-assisted group n=8 and conventional neuropsychological group therapy control n=8.	Two-group pre/post intervention study. Assessments included WISC-IV, Italian Battery for Evaluation of Dyslexia and Dysorthography, and Multidimensional Self-Esteem Test before and after treatment.	Donkey-assisted therapy improved self-esteem, reading performance, writing skills, and selected academic outcomes among children with dyslexia. The control group improved on some academic measures but not self-esteem.

Study ID	Country	Aims	Participants	Study design / data collection	Key findings[1]
					Authors suggested donkey-assisted therapy may enhance motivation and rehabilitation engagement.
De Rose et al. (2011)	Italy	To estimate the potential for donkey-assisted rehabilitation in children's outpatient rehabilitation units and to evaluate child-donkey approach modalities for designing a structured protocol.	Four children aged 6-12 years receiving logopedic/neuropsychomotor outpatient treatment with varied neurodevelopmental needs including language disturbance, ADHD traits, mild intellectual disability/neurodevelopmental disorder, and Down syndrome.	Pilot observational study. Children received weekly 45-minute donkey-assisted rehabilitation sessions for 6 months. Baseline, 3-month, and 6-month assessments included projective/drawing tasks, therapist observation sheets, and some video material.	Children relied more on physical expression than verbal language when communicating with donkeys; strong emotional participation was observed. Drawings suggested increased attention to reality, detail, and affective expression. Authors proposed donkey mediation as a complement to traditional rehabilitation.
Dicé et al. (2017)	Italy	To evaluate an animal-assisted education intervention designed to promote emotional recognition, emotional understanding, and relational skills among primary school children.	21 third-grade students (approximately 8 years old).	Preliminary observational animal-assisted education study conducted in a primary school. Intervention delivered by a psychologist and veterinarian trained in Human–Animal Interaction using one dog and three donkeys.	Donkey-assisted education promoted recognition of primary emotions, emotional expression, and relational skills among elementary school students.
Farm Animals and Livestock/Goats/Intervention Studies					
Harada et al. (2019)	Japan	To examine mood-related effects and participant attitudes during interactions with a stationed therapy goat among children, students, and older adults.	Participants included older adults in a nursing home, elementary and high school students, and preschool children. Number of children and their ages were not provided.	Clinical/field study using a trained therapy goat under a specialist handler. The study examined changes in mood and attitudes toward interacting with the goat across multiple age groups/settings with the goal	Participants reported favorable attitudes toward goats and improved mood and quality of life following interactions with therapy goats.

Study ID	Country	Aims	Participants	Study design / data collection	Key findings[1]
				of improving the quality of life (QOL) of the children	
Farm Animals and Livestock/Mixed/Intervention Studies					
Byström et al. (2022)	Sweden	To describe development in play, social communication, and mentalization in an 8-year-old boy with autism, mild intellectual disability, and major communication delays during and after COMSI, a nature- and animal-based group treatment.	Single case: 'William,' aged 8 years 9 months, selected from a five-child COMSI group because he completed treatment and had the lowest verbal ability.	Mixed-methods single-case study of a 1.5-year nature- and animal-based treatment on a small farm. Data included therapist process notes, parent interviews, WPPSI-III, TROG-2, CARS, and false-belief/mentalization tasks. Animals acted as a shared focus for communication.	The child showed positive development in play, social communication, and mentalization, including improved false-belief performance and increased verbal/nonverbal developmental estimates. Authors attributed change to coordinated support from animals, nature, and sensitive therapists.
Dolecheck et al. (2024)	USA	To explore parent perspectives regarding the effects of farm animal-assisted therapy for children with autism spectrum disorder.	Five parents participating in family-session farm animal assisted therapy programs for children with ASD.	Qualitative phenomenological study using semi-structured interviews. Intervention incorporated the Horse Boy Method and multiple farm animal species.	Parents reported improvements in social behaviors, behavioral regulation, communication, and a sense of normalcy. Findings suggest farm animal-assisted therapy may provide meaningful benefits for children with ASD and their families.
Geldhof et al. (2021)	USA	To examine whether classroom emotion regulation changed before and after animal-assisted activities and whether effects varied by student.	Nine sixth- and seventh-grade boys from one classroom at a mental health treatment facility/special education school for youth with persistent psychosocial and educational challenges.	Pilot evaluation using intensive observational data from 163 five-minute classroom video clips collected across weeks 2-25 of the academic year. Facial affect was coded using FACES; behavior around farm and wildlife AAI conditions was compared with active/passive control conditions	Whole-sample findings suggested anticipatory changes before AAI but no clear sample-level post-AAI regulation effect. Person-specific analyses showed effects and even measure validity varied substantially by student.

Study ID	Country	Aims	Participants	Study design / data collection	Key findings[1]
				using multilevel and person-specific models.	
Novotná et al. (2025)	Czech Republic	To evaluate whether outdoor learning in the presence of farm animals during animal-oriented lessons affects knowledge performance and memory retention in elementary school children.	149 fourth-grade pupils aged 9-11 years from four public elementary schools after exclusion of 31 pupils with incomplete testing; experimental n=71 and control n=78.	Exploratory controlled educational study. Experimental group attended a 90-minute eco-center farm program including 45 minutes of teaching with direct animal exposure; control group received identical content in a 45-minute classroom lesson using conventional aids. Knowledge was tested pre, immediately post, and 28 days later.	Farm-animal-assisted education improved long-term memory retention compared with conventional classroom instruction.
Torquati et al. (2025)	Italy	To test the feasibility, preliminary effects, animal-welfare profile, and economic sustainability of cow-assisted interventions in social farming.	Three participant groups: adolescents removed from families, young adults with mental health disorders, and individuals with eating disorders. Four Red Pied Valdostana cows were used.	Pilot social-farming study with structured cow-assisted sessions including observation, feeding, grooming, problem solving, and leading. Human outcomes were assessed across emotional, relational, and behavioral dimensions; animal welfare was monitored continuously; costs were analyzed for animal assisted activities, animal-assisted education, and animal assisted therapy formats.	Participants reported improved self-esteem, emotional expression, and social interaction; the eating-disorder group showed greater openness toward dairy consumption. Cow welfare remained stable, with high tolerance to handling. Staff costs were the main cost driver; reported hourly costs were €74.51 for animal assisted activities, €144.99 for animal-assisted education, and €172.41 for animal assisted therapy.
Fish and Aquatic Species/Intervention Studies					
Sarman & Günay (2023)	Türkiye	To determine the effect of a goldfish intervention on anxiety, fear,	112 hospitalized children aged 8-10 years in Türkiye's Eastern Anatolia	Open-label, single-center randomized controlled trial conducted	Goldfish intervention significantly reduced anxiety and fear while

Study ID	Country	Aims	Participants	Study design / data collection	Key findings[1]
		and psychological and emotional well-being among hospitalized children.	region; intervention n=56, control n=56.	November 2020-August 2021. Children in the study group observed/cared for goldfish for 3 days. Measures included State-Trait Anxiety Inventory for Children, Child Fear Scale, and Stirling Children's Well-being Scale.	improving psychological and emotional well-being among hospitalized children compared with controls.
Sarman & Tuncay (2024)	Türkiye	To compare goldfish and aquatic turtle interventions for reducing children's pain, anxiety, and fear during intravenous catheter insertion.	98 children aged 5-6 years attending an outpatient clinic for blood sampling and catheterization; control n=32, goldfish n=33, aquatic turtle n=33.	Randomized controlled trial with three arms. Pain was measured with the Wong-Baker Facial Pain Scale, anxiety with the Child Anxiety Scale-State, and fear with the Child Fear Scale before, during, and after catheterization.	Goldfish and aquatic turtle interventions reduced pain, anxiety, and fear during intravenous catheterization, with anxiety and fear remaining significantly lower after the procedure.
Yilmaz & Demir Acar (2024)	Türkiye	To determine whether a goldfish-based animal-assisted intervention reduced fear among children hospitalized in a pediatric emergency department.	70 children aged 5-7 years hospitalized in a pediatric emergency department in the Central Black Sea Region of Türkiye; intervention n=35, control n=35.	Randomized controlled trial conducted April-June 2023. Children in the intervention group interacted with a goldfish during ED hospitalization while controls received routine care. Measures included the Child Fear Scale and the Scale of Children's Fear of Nursing Interventions and Instruments Used in Hospital.	Goldfish-assisted intervention significantly reduced fear during emergency department hospitalization and decreased fear related to nursing procedures and hospital equipment.
Exotic and Wildlife Species/Elephant/Intervention Studies					
Satiansukpong et al. (2016)	Thailand	To examine the effects of the Thai Elephant-Assisted Therapy Programme for children with Down	16 children with Down syndrome in grades 1-6 at a public school in Thailand; participants voluntarily	Quasi-experimental design with blinded control. Both groups received regular school activities; the	Elephant-assisted therapy significantly improved visual-motor integration among children with Down

Study ID	Country	Aims	Participants	Study design / data collection	Key findings[1]
		syndrome on balance, postural control, and visual-motor integration.	allocated to control and experimental groups.	experimental group additionally received Thai elephant-assisted therapy twice weekly for 2 months. Measures included BOT-2 balance subtest, postural control record form, and Beery VMI before and after the program.	syndrome; no significant changes were observed in balance or postural control.
Exotic and Wildlife Species/Reptiles/Intervention Studies					
Murry & Allen (2012)	USA	To evaluate whether reptile-assisted support reduced internalizing, externalizing, and other behavioral problems among female children with emotional disturbance who had refused traditional talk therapy.	40 twelve-year-old female children with emotional disturbance who had experienced the death of a parent in the preceding year; after withdrawal, analyzed n=16 reptile-assisted and n=19 control.	Randomized pre-post support-group comparison. Groups met weekly for 16 weeks for 75 minutes. Reptile-assisted groups discussed death/grief while learning reptile care and emotion-related interpretation; controls discussed death/grief without reptile reference. Primary caregivers completed CBCL-P before and after.	Reptile-assisted support was associated with improved internalizing scores, especially somatic behaviors, externalizing scores, especially delinquency, and social, thought, and attention behaviors. Total CBCL-P scores improved in both groups, with no significant difference, but reptile-assisted participants showed stronger change on several subscales. Anxious/depressed behaviors did not significantly improve.
Exotic and Wildlife Species/Invertebrates/Intervention Studies					
Kim et al. (2018)	Republic of Korea	To investigate effects of rearing pet insects on mental health and diurnal salivary cortisol profiles in mentally healthy elementary school children.	38 elementary school pupils from grade 4-5 without psychological disorders; 19 boys and 19 girls; mean age 11.97 years.	Pilot pre/post classroom program over 8 weeks. Pupils selected and reared three types of insects from six options. Education sessions occurred every two weeks. Measures included salivary cortisol four times	Rearing insects improved self-esteem, reduced daily stress, and was associated with favorable changes in diurnal salivary cortisol profiles; no significant changes were

Study ID	Country	Aims	Participants	Study design / data collection	Key findings[1]
				daily, Attention Deficiency and Hyperactivity scale, self-esteem, and daily hassles.	observed in attention-deficit/hyperactivity symptoms.
Exotic and Wildlife Species/Invertebrates/Educational and Human-Animal Relationship Studies					
Hirschenhauser & Brodesser (2023)	Austria	To examine children's relationships with a Giant African land snail in a school setting and explore implications for educational and therapeutic applications of non-vertebrate species	15 seven-year-old primary school children exposed to a classroom Giant African land snail for seven months.	Exploratory school-based study assessing children's attachment and caregiving relationships toward a classroom snail.	Children demonstrated moderate-to-high relationship scores with the snail, comparable to relationships reported with dogs, cats, and rabbits. Findings suggest non-vertebrate species may have potential in educational and therapeutic contexts.
Multi-Species Programs/Intervention Studies					
Conniff et al. (2005)	USA	To assess whether an ongoing pet-visitation animal-assisted activity program affected behavior and emotional state of adjudicated female adolescents in a medium-secure residential facility.	23 adjudicated female adolescents aged 13-17 years; randomly assigned to pet visitation n=13 or usual activities n=10; attrition left 12 and 6 respectively for analysis.	Randomized pretest-posttest pilot over 8 weeks. Cornell Companions teams visited weekly for one-hour unstructured sessions. Outcomes included Youth Self-Report, Resident Behavior Assessment, and a qualitative survey.	Quantitative measures did not demonstrate significant behavioral or emotional changes; however, participants reported enjoying the pet visitation program and perceived it positively.
Flynn et al. (2024)	USA	To examine how relationships with animals function as ecological assets for youth development and identify processes through which AAI may influence psychological, social-emotional, and developmental outcomes among students	30 students (ages approximately 10–14 years) enrolled in a special education and mental health treatment program incorporating AAI with multiple species in a farm setting.	Qualitative phenomenological study using semi-structured interviews with students participating in animal-assisted programs.	Students described animals as promoting social connection, confidence, enjoyment, self-regulation, and positive emotional experiences. Relationships with animals were viewed as important developmental assets.

Study ID	Country	Aims	Participants	Study design / data collection	Key findings[1]
		receiving special education and mental health services.			
Locher et al. (2023)	Switzer-land	To examine feasibility and potential efficacy of animal-assisted group psychotherapy for pediatric chronic pain.	Three girls aged 9-12 years with chronic head and abdominal pain; recruitment was via psychosomatic and pain consultations.	Open pilot case series with mixed methods. The intervention was group-based animal-assisted psychotherapy at a farm/nature setting. Quantitative pre/post measures focused on pain severity, pain-related disability/distress, pain acceptance, avoidance/fear of pain, engagement in activities, and defocusing; qualitative interviews captured expectations and experiences.	Recruitment was challenging, partly due to COVID-19. All three children reported reduced pain-related disability and distress and improved pain acceptance/defocusing. Families had positive attitudes toward AAI. Authors called for randomized controlled trials with larger samples.
Mudaly et al. (2014)	Australia	To obtain children's perspectives on benefits of an animal-assisted education and therapy group for homeless children traumatized by family violence.	Children and young people who had attended an animal therapy group run by a family-violence housing/support agency in Melbourne; 11 children total; 6 boys, 5 girls ages 7-15).	Preliminary qualitative evaluation commissioned by the service agency. Child-centered interviews/dialogue captured participants' views of an established group-based animal-assisted education program using small animals and supported by case management.	Children described feeling calmer when holding animals, improved emotion regulation, safer engagement, and benefits for anger and coping. The evaluation emphasized acceptability and perceived benefits rather than quantitative efficacy.
Sokal & Kahl (2019)	Germany	To examine whether duration and type of school-based animal-assisted activities were associated with perceived mental well-being	Minority students at a German boarding school, described as largely Islamic/migrant students. 13 students in grades 5 to 9 for each of rabbit club and dog club (rabbit	Mixed-methods school-based animal assisted activities evaluation using surveys, interviews, and observations. Short-term calmness and longer-term well-being were	Short-term gains in calmness occurred under some animal assisted activity conditions, and long-term gains in well-being were reported for students participating in rabbit

Study ID	Country	Aims	Participants	Study design / data collection	Key findings[1]
		among minority students at a boarding school in Germany.	n = 13; control n = 13); rabbit club students were self-nominated.	examined across animal assisted activities conditions including rabbit club and dog club.	club. Overall well-being effects were significant, but self-regulation, social cohesion, and social support effects were not significant. Rabbit-club gains were not replicated in dog club.
Unidentified Species/Intervention Studies					
Tahan et al. (2022)	Iran	To assess the effectiveness of animal-assisted therapy in reducing anxiety symptoms in preschool children.	20 anxious children aged 5-7 years selected from a welfare anxiety screening program in Tehran, Iran and based on having an anxiety score higher than the average score; randomized to experimental a animal assisted therapy (n=10) or no-intervention control (n=10).	Randomized controlled trial with pretest-posttest design. The experimental group received eight 90-minute animal assisted therapy sessions; controls received no intervention. Mothers completed the Spence Preschool Anxiety Scale (Parent Form), and DSM-V based structured interviews were conducted before and after treatment. ANCOVA was used.	Animal assisted therapy significantly reduced general anxiety after adjustment (F=32.49, p=0.001, effect=0.70). Significant effects were also reported for separation anxiety, generalized anxiety disorder, social phobia, and specific anxiety; effects on obsessive symptoms were not significant.

[1]Key findings summarize results as reported by the original study authors. They should not be interpreted as independent conclusions of the present scoping review regarding intervention efficacy, effectiveness, or causality. Formal study-level risk-of-bias assessment was not conducted.
